# Thymosin β4 increases cardiac cell proliferation, cell engraftment, and the reparative potency of human induced-pluripotent stem cell-derived cardiomyocytes in a porcine model of acute myocardial infarction

**DOI:** 10.7150/thno.56757

**Published:** 2021-06-26

**Authors:** Shi Hua Tan, Sze Jie Loo, Yu Gao, Zhong Hao Tao, Li Ping Su, Chen Xu Wang, Sophia L. Zhang, Yong Hui Mu, Ying Hua Cui, Desiree Abdurrachim, Wei Hsin Wang, Janise Lalic, Kheng Choon Lim, Jun Bu, Ru San Tan, Teck Hock Lee, Jianyi Zhang, Lei Ye

**Affiliations:** 1 National Heart Research Institute Singapore, National Heart Centre Singapore, Singapore.; 2 Department of Cardiology, Ren Ji Hospital, School of Medicine, Shanghai Jiao Tong University, Shanghai 200127, China.; 3 Department of Thoracic and Cardiovascular Surgery, Nanjing First Hospital, Nanjing Medical University, Nanjing, Jiangsu, China.; 4 Xinxiang Medical University, Xinxiang, Henan, China.; 5 Singapore Bioimaging Consortium, A*STAR, Singapore.; 6 Department of Diagnostic Radiology, Singapore General Hospital, Singapore.; 7 Department of Biomedical Engineering, The University of Alabama at Birmingham, Birmingham, AL, USA.

**Keywords:** Myocardial infarction, Stem cell therapy, Microsphere.

## Abstract

**Rationale:** Previous studies have shown that human embryonic stem cell-derived cardiomyocytes improved myocardial recovery when administered to infarcted pig and non-human primate hearts. However, the engraftment of intramyocardially delivered cells is poor and the effectiveness of clinically relevant doses of human induced pluripotent stem cell-derived cardiomyocytes (hiPSC-CMs) in large animal models of myocardial injury remains unknown. Here, we determined whether thymosin β4 (Tb4) could improve the engraftment and reparative potency of transplanted hiPSC-CMs in a porcine model of myocardial infarction (MI).

**Methods:** Tb4 was delivered from injected gelatin microspheres, which extended the duration of Tb4 administration for up to two weeks *in vitro*. After MI induction, pigs were randomly distributed into 4 treatment groups: the MI Group was injected with basal medium; the Tb4 Group received gelatin microspheres carrying Tb4; the CM Group was treated with 1.2 × 10^8^ hiPSC-CMs; and the Tb4+CM Group received both the Tb4 microspheres and hiPSC-CMs. Myocardial recovery was assessed by cardiac magnetic resonance imaging (MRI), arrhythmogenesis was monitored with implanted loop recorders, and tumorigenesis was evaluated via whole-body MRI.

**Results**: *In vitro*, 600 ng/mL of Tb4 protected cultured hiPSC-CMs from hypoxic damage by upregulating AKT activity and BcL-XL and promoted hiPSC-CM and hiPSC-EC proliferation. In infarcted pig hearts, hiPSC-CM transplantation alone had a minimal effect on myocardial recovery, but co-treatment with Tb4 significantly enhanced hiPSC-CM engraftment, induced vasculogenesis and the proliferation of cardiomyocytes and endothelial cells, improved left ventricular systolic function, and reduced infarct size. hiPSC-CM implantation did not increase incidence of ventricular arrhythmia and did not induce tumorigenesis in the immunosuppressed pigs.

**Conclusions**: Co-treatment with Tb4-microspheres and hiPSC-CMs was safe and enhanced the reparative potency of hiPSC-CMs for myocardial repair in a large-animal model of MI.

## Introduction

Ischemia results in the loss of cardiomyocytes (CMs), functional blood vessels, and perfusion in the infarcted myocardium. The ability of the heart to regenerate after ischemic injury is very limited; thus, cellular cardiomyoplasty is being investigated as a method for repairing or regenerating ischemic myocardium. Large quantities of human CMs have been successfully generated from human embryonic stem cells (hESCs) and induced-pluripotent stem cells (hiPSCs) (collectively referred to as human pluripotent stem cells [hPSCs]) via a variety of differentiation protocols 1-3 and their effectiveness for cardiac repair is being tested in numerous animal models 4. Although hESC-derived CMs (hESC-CMs) improved myocardial performance in infarcted rodent hearts 5-7, there are conflicting results from large-animal investigations of hPSC-CMs and CMs derived from monkey induced-pluripotent stem cells (miPSC-CMs) 4, 8-10. Liu and Shiba, et al., demonstrated that transplanted hESC-CMs or miPSC-CMs improved heart function in non-human primate models of myocardial infarction (MI) 8, 10, but hESC-CMs did not improve measures of cardiac function at 4 weeks after administration to infarcted pig hearts, despite evidence of myocardial regeneration 9.

Previously, we have shown that in pigs with acute MI, fibrin patches containing insulin-like growth factor 1 (IGF-1) with hiPSC-derived endothelial cells (ECs), smooth muscle cells (SMCs), and CMs transplantation, led to significant improvements in left ventricular (LV) function, myocardial metabolism, arteriole density, infarct size, LV wall stress, and apoptosis 4, which suggest that the effectiveness of hiPSC-CMs for cellular cardiomyoplasty may be enhanced by the addition of other beneficial factors.

The actin-sequestering protein thymosin β4 (Tb4) is known to improve the survival, proliferation, and migration of cardiac cells 11, to promote cardioprotection 11-14, angiogenesis, and vasculogenesis 15-17, and to improve cell engraftment 18, as well as the differentiation and vascularization of engineered heart tissue 19. Thus, our current study combines the controlled release of Tb4 with hiPSC-CMs for treatment of acute MI. Furthermore, clinical translation requires assessments of efficacy and safety in large-animals with hearts that closely resemble those of humans. Although studies with clinically relevant numbers of hESC-CMs and miPSC-CMs have been conducted in pigs 9 and non-human primates 8, 10, 20, similar studies have not been reported with hiPSC-CMs. Therefore, our experiments aim to determine the efficacy and safety (e.g., arrhythmogenic and tumorigenic potential) of a clinically relevant number of hiPSC-CMs (1.2
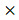
10^8^) with or without the controlled release of Tb4 for treatment of acute MI in pigs, whose hearts resemble human hearts in both size and weight. We expect our results to support further investigations of cryo-preserved allogeneic hiPSC-CMs and Tb4-containing gelatin microspheres as an adjunctive therapy with coronary artery bypass surgery for the treatment of acute MI.

## Methods

### Culture and differentiation of human induced pluripotent stem cells

An established hiPSC line, PCBC, was used in the current study 21, 22. hiPSCs were cultured as a monolayer on growth factor reduced Matrigel coated surface in mTeSR/E8 (1 : 1) media (both from Stem Cell Tech., Canada). The differentiation protocol of hiPSCs into CMs had been described 23. Generally, differentiated hiPSCs would start contracting between day 8-10 of differentiation. The purity of hiPSC-CMs was assessed by fluorescence staining for cardiac Troponin T (cTnT) (564766, BD Biosciences, USA) expression using flow cytometry 21. hiPSC-CMs used in this study were immature CMs which expressed cTnT and atrial myosin light chain 2 (MLC2a), but very lower level of cardiac Troponin I3 (TNNI3) and did not express ventricular myosin light chain 2 (MLC2v) protein 24.

### Purification of hiPSC-CMs

Purified hiPSC-CMs were used for *in vitro* studies. The purification protocol was described previously 21. Briefly, hiPSC-CMs were dissociated into single cells on day 7 of contraction and cultured in RPMI1640 medium without glucose (RPMI-) supplemented with 1 x non-essential amino acids, 1 x L-Glutamine GlutaMAX, 1 x Antibiotic-Antimycotic, 55 nM β-mercaptoethanol (all from Thermo Fisher, USA), and 4 mM lactic acid (Sigma Aldrich, USA) for 6 days.

### Profile of angiogenic factors released from hiPSC-CMs

For assessment of angiogenic factors released from Tb4 non-treated or treated hiPSC-CMs, 2 x 10^5^ hiPSC-CMs/well were seeded in 12-well plate and cultured in 1 mL RPMI medium supplemented with 50 x dilution of B27 with or without 600 ng/mL Tb4 for 48 h. The supernatant was collected and used to determine the profiles of angiogenic factors released from hiPSC-CM using Human Angiogenesis Arrays Q2 and Q3 kits as described 4.

### *In vitro* assessments

#### Hypoxia treatment

For assessment of cyto-protection by Tb4 on hiPSC-CMs, 2 x 10^5^ hiPSC-CMs/well were cultured in 12-well plate. After washing thrice with Dulbecco's phosphate-buffered saline (DPBS), hiPSC-CMs were cultured in 500 μL Hanks balanced salt solution (HBSS) supplemented with or without Tb4 protein (Prospec, USA) and cultured in an incubator with hypoxic condition for 24 h: 5% CO_2_, 94% N_2_, and 1% O_2._

#### Lactate dehydrogenase (LDH) assay and DNA damage measurement

The supernatant was collected to determine the intensity of LDH fluorescence in the supernatant using the Cytotoxicity detection kit (Roche, USA) per manufacturer's instructions 24. A human DNA fragmentation factor subunit beta ELISA kit (Abbexa, USA) was used to determine damaged DNA released into the supernatant according to manufacturer's instructions.

#### Terminal deoxynucleotidyl transferase dUTP Nick-End Labeling (TUNEL) assay

Human iPSC-CMs underwent hypoxia were used for assessment of apoptosis using an *In situ* Cell Death Detection Kit (Roche Applied Science, Germany) as described 18. Apoptotic cells were evaluated by counting the number of TUNEL^+^CM nuclei/total cell nuclei per field. Experiments were repeated thrice.

#### Western Blot analysis

Total protein was isolated using PhosphoSafe™ Extraction Reagent (Merck, Germany) and protein concentration was determined using Bradford reagent (Bio-Rad Laboratories, USA) per manufacturer's instruction 25. Proteins (3 µg/sample) were separated on SDS-polyacrylamide gel and were transferred onto nitrocellulose membrane. After blocking with 5% non-fat milk in Tris-buffered saline Tween-20 buffer (25 mM Tris, pH 7.5, 150 mM NaCl, and 0.1% Tween-20), the blots were incubated with primary antibodies: rabbit anti-glyceraldehyde phosphate dehydrogenase (GAPDH) at 1: 5000 dilution; p-AKT (S473) at 1: 2000 dilution; AKT at 1: 2000 dilution; and Bcl-XL at 1: 1000 dilution (all from Cell Signaling, USA). Anti-rabbit IgG conjugated with horseradish peroxidase (1: 5000 for GAPDH and 1: 4000 for the rest) was used to detect the binding of antibodies. The binding of the specific antibody was detected using the SuperSignal Chemiluminescent Substrate kit (Pierce, USA) and visualized using ChemiDoc™ XRS+ System (Bio-Rad, USA). The protein expression level was normalized by GAPDH and expressed as percentage of GAPDH.

### Proliferation of Cardiac cells in response to Tb4

For assessing CM proliferation, 2x10^4^ hiPSC-CMs were seeded in wells of 16-well chamber slides and cultured in RPMI supplemented with 50 x dilution of B27 and with or without 600 ng/mL Tb4 for 4 days. Medium was changed every 2 days. Then, cells were co-stained for protein expression of Aurora B Kinase (ABK, ab2254, Abcam, USA) or Ki67 (MA5-14520, Thermo Fisher, USA) with cTnT (T8665-23A, USBiological Life Sciences, USA).

For assessing endothelial cell (EC) and smooth muscle cell (SMC) proliferation, 4x10^5^ hiPSC-CMs were seeded into wells of 6-well plate and were either cultured in endothelial basal medium (EBM, Lonza, Switzerland) or M231 medium (a basal medium for SMC) (Thermo Fisher, USA) for 48 h to obtain conditioned EBM (cEBM) or M231 medium (cM231). 5x10^4^ hiPSCs differentiated ECs (hiPSC-ECs) 22 were seeded in wells of 16-well chamber slides and were cultured in cEBM, or fresh EBM (fEBM) supplemented with 600 ng/mL of Tb4, or cEBM supplemented with 600 ng/mL Tb4 for 48 h. 4x10^4^ human coronary artery SMCs (Thermo Fisher, USA) were seeded in wells of 16-well chamber slides and were cultured in cM231, or fresh M231 (fM231) supplemented with 600 ng/mL Tb4, or cM231 supplemented with 600 ng/mL Tb4 for 24 h. 100x dilution of B27 was added into respective cell cultured medium. After treatment, cells were co-stained for protein expression of Ki67 and CD31 (BD, USA) or smooth muscle actin (SMA) (A2547, Sigma-Aldrich, USA).

### Gelatin microspheres for controlled release of Tb4

Gelatin microspheres were manufactured as described with modifications 18. Briefly, 5 mL of 10% gelatin (type A, Sigma-Aldrich, USA) solution at 50 °C was added into 45 °C olive, stirred, and cooled to 5 °C. 25 min later, chilled (4 °C) acetone was added to the olive oil to induce microsphere formation. Then, microspheres were collected, washed 5 times to remove the olive oil, air-dried at 4 °C, and resuspended in chilled (4 °C) 70% ethanol containing 1% glutaraldehyde (Sigma-Aldrich, USA) to induce cross-linking for 30 min at 4 °C. The mixture was neutralized with an equal volume of 0.1 M glycine (Sigma-Aldrich, USA). Cross-linked microspheres were collected by washing with ethanol solution and air-dried. Tb4 (Prospec, USA) was loaded into the microspheres by mixing 25 mg microspheres with 25 µL distilled H_2_O containing 25 µg Tb4.

To determine the rate of Tb4 released from the microspheres *in vitro*, 2 mL RPMI and 25 mg microspheres containing 25 μg Tb4 were added to each well of 6-well plates. 1 mL of the conditioned medium was collected and replaced with fresh RPMI on day 1 and every other day afterwards for 14 days. The concentration of Tb4 in the conditioned medium was determined using a Tb4 ELISA kit (Peninsula Laboratories International, USA) as directed by the manufacturer's instructions.

To determine whether Tb4 would promote hiPSC-CM maturation, hiPSC-CMs were cultured in RPMI medium supplement with 50 x dilution of B27 and with or without 600 ng/mL of Tb4 for 14 days. Medium was changed every 2 days. Cells were collected and used for fluorescence immunostaining to determine protein expressions of MLC2a (Proteintech Group, USA), MLC2v (Proteintech Group, USA), cTnT (USBiological Life Sciences, USA), and TNNI3 (Abcam, USA) as described 24.

To determine the duration of the Tb4 release *in vivo*, 3 μg Tb4-his-tag (Tb4-his, P5500, Wuhan Fine Biotech., China) were loaded into 3 mg microspheres and injected into rat hearts. The animal experimental protocol was approved by the Institutional Animal Care and Use Committee. Animal experimental procedures were performed in accordance with the Animal Use Guidelines, of the Singapore Health Services Pte Ltd. Four female rats (~180 g) were anesthetized with intraperitoneal injection of 0.1 mL/100g body weight of Ketamine/ xylazine mixture (1 mL containing 50 mg ketamine and 20 mg xyzaline). 100 μl RMPI containing Tb4-his loaded microspheres were injected into left ventricular anterior wall. All animals received cyclosporine injection (15 mg/kg) daily until 2 weeks after treatment. Rat blood was collected from tail vein on days 2, 4, 6, 9, 11, 14 after surgery. Serum was isolated by centrifuge and stored in -80°C freezer until analysis. Rats were euthanized on day 14 after surgery. Left ventricular anterior walls were isolated, weighed, grinded in liquid nitrogen, and further lysed using Zirconia beads (11079110zx, Biospec., USA) and Bullet Blender Homogenizer (NextAdvance, USA). The concentration of his-tag in serum and heart tissue lysate were determined using a His Tag ELISA Detection Kit (L00436, GenScript Biotech, USA). The concentration or quantity of Tb4-his in serum or heart tissue was calculated as the concentration of his-tag divided by 0.12, as the concentration of his-tag in recombinant Tb4-his is 12%.

### Toxicological assay of gelatin microspheres on cardiac cells *in vitro*

To determine whether gelatin microspheres had cytotoxic effect on cardiac cells, hiPSC-CM, hiPSC-ECs 22, and SMCs were seeded at 1 x 10^5^, 5 x 10^4^, and 5 x 10^4^, respectively, in wells of 12-well plate. Cells were cultured in their respective medium: hiPSC-CM: RMPI + 50 x dilution of B27, hiPSC-ECs: endothelial growth medium (EGM, Lonza, Switzerland) + 10 μM SB431542 (Selleckchem, USA) +100 ng/mL vascular endothelial growth factor-A (VEGF-A, ABM, Canada), and SMC: M231 supplemented with smooth muscle growth supplement (Thermo Fisher, USA). Supernatant was collected every 2 days for measurement of the intensity of LDH as described above 18, 24.

### Pig heart models of MI and cell transplantation

The animal experimental protocol was approved by the Institutional Animal Care and Use Committee. Animal experimental procedures were performed in accordance with the Animal Use Guidelines, of the Singapore Health Services Pte Ltd. Experiments were performed on Yorkshire-landrace swine (~13 kg body weight). Coronary arteries of 44 pigs were permanently ligated as described previously 4, 26, 27. Briefly, pigs were sedated with an intramuscular injection of 1 mL/10 Kg body weight of ketamine (100 mg/mL)/Xylazine (20 mg/mL) mixture. Anesthesia was maintained with 2 - 2.5% isoflurane on a ventilator after intubation. A limited left lateral thoracotomy was performed to expose the heart. The 1^st^ branches of left anterior descending and left circumflex coronary arteries were permanently ligated to create an infarction. Then the chest was closed. This consistently induced an infarct size of 14% of the LV wall. All animals received analgesia (Ketoprofen 5 mg/kg/day), and antibiotics (Enrofloxacin 15 mg/kg) after surgery.

Four pigs died immediately after coronary artery ligation. The remaining pigs (n=40) were randomly assigned into four groups and received treatment immediately after MI (Table S1): the MI Group (n=8) received 1 mL RPMI injection; the Tb4 Group (n=9): received 1 mL RPMI containing 25 mg of gelatin microspheres loaded with 25 μg Tb4 (Tb4-microspheres); the CM Group (n=8): received 1 mL RPMI containing hiPSC-CMs on day 7 after starting contraction; and the Tb4+CM Group (n=8): received 1 mL RPMI containing Tb4-microspheres + hiPSC-CMs on day 7 after starting contraction (Table S1). One mL RPMI without or with Tb4-microspheres, or hiPSC-CMs, or Tb4-microspheres + hiPSC-CMs were intramyocardially injected (10 injections) into the center and border zone of infarct through a fibrin patch which was applied epicardially on the infarcted myocardium 4. An additional seven pigs were added to the Tb4 + CM Group to be followed-up to 12 weeks after MI to determine tumor formation in major organs using whole body MRI and one animal died during MRI study. Another eight pigs underwent sham surgery and served as the Sham Group. All pigs were fed with cyclosporin (15 mg/ Kg of body weight) starting 3 days before MI surgery until the day of euthanization.

In preparation for cell implantation, hiPSC-CMs on day 7 after starting contraction were harvested, resuspended in StemMACS™ Cryo-Brew freezing medium (Miltenyi Biotec, Germany), and stored in liquid nitrogen. On the day of cell transplantation, 1.2
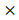
10^8^ differentiated hiPSC-CMs were recovered and resuspended in 1 mL of RPMI and intramyocardially injected into the border and infarct zones of MI through a fibrin/thrombin patch applied on the epicardium of the infarcted myocardium 4. A total of 10 injections were performed and each injection contained about 1.2 x 10^7^ cells. The viability of cells was determined by trypan blue staining.

### Cardiac magnetic resonance imaging

Cardiac MRI was performed at 1, 4, and 12 weeks (for pigs with 12-week follow-up) after MI and treatment using a 3.0 T whole body MRI machine (Siemens Skyra, Siemens Medical Systems, Erlangen, Germany) with a standard cardiac flex coil (Siemens Medical Systems, Erlangen, Germany) as described 27. MRI imaging was performed by staff who was blinded to the animal groups. Pigs were sedated with intramuscular injection of ketamine/xylazine mixture. Anesthesia was maintained with 2 - 2.5% isoflurane on a ventilator after intubation. Heart rate and oxygen saturation were monitored throughout the examination. LV function was investigated using a segmented breath-held steady state free precession cine MRI imaging sequence. Contiguous 10 to 12 short-axis 2D slices without gap covering the LV from base to apex were acquired. Typical parameters were: TR/TE = 48.6 ms/ 2.27 ms, matrix = 128 x 128, FOV = 81 mm x 81 mm, flip angle = 40°, slice thickness = 4 mm, ECG gated, number of phases = 8 - 10. Gadolinium-based contrast agent Magnevist (0.3 mmol/Kg, gadopentetate dimeglumine, Bayer Healthcare, Bayer) was injected intravenously. After 10 min, late gadolinium enhancement inversion-recovery gradient echo sequences were acquired at the same positions as the cine images with the following parameters: TR/TE = 628.5 ms/ 2.35 ms, TI = 350 ms, matrix = 128 x 128, FOV = 81 mm x 81 mm, flip angle = 30°, slice thickness = 4 mm, ECG gated.

Global function was computed from the short-axis cine images by semi-automated segmentation of the LV endocardial and epicardial borders (from base to apex) at both end-diastole and end-systole using CVi42 analysis software (Circle Cardiovascular Imaging Inc., Canada). Infarct size was calculated from the late gadolinium enhancement images with scar surface area expressed as a percentage of the total LV surface area 4, 27*.* Briefly, within the defined endocardial and epicardial borders, the LV area with signal intensity > mean + 2 SD of non-infarcted septal wall intensity, was considered as scar. The analysis of MRI data was not blinded.

### Real-time electrocardiogram monitoring

The implantable loop recorders (Medtronic-Reveal, USA) were subcutaneously placed in the left paraspinal area inferior to the angle of the scapula in pigs after surgery. The recorded ECG data from the loop recorders were transmitted to a server once every two days. The incidences of ventricular arrhythmia, including atrioventricular dissociation (AVD) and ventricular tachycardia (VT) were calculated.

### Whole body MRI for tumor detection

Whole body MRI was performed on 7 pigs from the Tb4 + CM Group at weeks 1 and 12 after MI and treatment using a 3.0 T whole body clinical MRI (Siemens Skyra, Siemens Medical Systems, Erlangen, Germany). Pigs were sedated with intramuscular injection of ketamine/xylazine mixture and anesthesia was maintained with 2 - 2.5% isoflurane on a ventilator after intubation. Animals were placed in supine position. To screen for any tumor formation in major organs, including limb, liver, pancreas, kidney, stomach, chest, and brain, whole body MRI was performed using the combination of body flex coils (18 channels) and spine coils. The whole-body scans consisted of four separate scan regions: (1) head, neck, and upper limb, (2) thorax, (3) abdomen, and (4) pelvis and lower limbs. Regions 1 and 4 were acquired using T2-weighted fat-suppressed fast-spin echo sequence, while regions 2 and 3 were acquired using T2-weighted short tau inversion recovery fat-suppressed turbo spin echo with breath-hold. The imaging parameters were as follows: TR/TE: 3850 ms/85 ms (region 1), 3340 ms/67 ms (regions 2 and 3), 3160 ms/90 ms (region 4), echo train length: 22 (regions 1 and 4) or 26 (regions 2 and 3), slice thickness: 5 mm, slice spacing: 3 mm, spatial resolution: ~1 mm. Total scan time was 25-30 min. The images were interpreted by a radiologist on RadiAnt DICOM Viewer [Software]. (Medixant, Version 4.0.3. Jun 10, 2017. URL: https://www.radiantviewer.com).

### Tissue harvest

All animals were euthanized at 4 or 12 (for 12-week follow-up) weeks after MI and treatment. Pig hearts were arrested by injecting 100 mg/ Kg KCl under anesthesia and explanted. The LV free wall was cut into 5 short-axis rings (R1-R5), and each ring was sequentially cut into 8 or 9 samples (S1-S9) 28. S3 and S4 of R3 (the center and border zone of cell transplanted area) were embedded in OCT or paraffin for immunohistochemistry studies.

### Immunohistochemistry

Vasculogenesis was evaluated by staining cryo-sections with rabbit anti-pig CD31 primary anti-body (ab28364, Abcam, USA), mouse IgG anti-SMA, and mouse IgM anti-α-sarcomere actin (α-SA) (A2172, Sigma, USA). Secondary antibodies, FITC-conjugated donkey anti-rabbit IgG, TRITC-conjugated donkey anti-mouse IgG, and Cy5-conjugated donkey anti-mouse IgM (all from Thermo Fisher, USA), were applied to visualize vessels in myocardium. Vascular structures positive for CD31 expression (FITC fluorescence), and for both CD31 and SMA expression (simultaneous FITC and TRITC fluorescence) were counted in 8-9 animals per group, in 4-5 slides per animal and 6-8 fields per slide at 200 x magnification using Olympus IX73 microscope and Cell Sens Standard software (Olympus, Japan).

Cadiomyocyte proliferation in animal heart was determined using paraffin sections. CM cytokinesis was determined by dual immunostaining for rabbit anti-ABK and mouse anti-cTnT. CM mitosis was determined by dual immunostaining for anti-Ki67 and cTnT. CM cytokinesis was quantified as the number of cTnT^+^CM expressing ABK with disassembled sarcomere and cleavage furrow per mm^2^ 27. CM mitosis was quantified as the number of cTnT^+^CM expressing Ki67 per mm^2^.

To quantify EC proliferation, cryo-sections of pig heart tissues were used. Tissue sections were dually stained with mouse anti-pig CD31 (MCA1746GA, BioRad-USA) and rabbit anti-Ki67. To quantify SMC proliferation, paraffin-sections of pig heart tissues were used. Tissue sections were dually stained with mouse anti-SMA and rabbit anti-Ki67. Proliferating EC or SMC was quantified as the number of CD31^+^ or SMA^+^ cells expressing Ki67 per mm^2^. Immunostaining images were acquired in a blinded manner and analysis was not blinded.

To determine immune response after MI and treatment, paraffin sections were dually stained with rabbit anti-pig CD11b (Abcam, USA) and mouse anti-cTnT. Number of CD11b^+^cells were counted in each stained tissue section. CD11b^+^cell density was quantified as the number of CD11b^+^cells per mm^2^ of tissue section.

### Engraftment of hiPSC-CMs in the porcine heart

The survival of transplanted hiPSC-CMs was evaluated by staining paraffin sections for mouse IgG anti-human nuclei (MAB1281, Millipore-Sigma, USA) and rabbit anti-cTnT to detect hiPSC-CMs in the pig hearts. Secondary antibodies, FITC conjugated donkey anti-mouse IgG or TRITC-conjugated donkey anti-rabbit IgG was applied to visualize CMs in the porcine heart. In addition, pig heart tissue sections were stained with mouse IgG anti-human nuclei, rabbit anti-connexin43 (SC9059, Santa Cruz Biotech, USA), and mouse anti-α-SA to determine the connexin43 protein expression between donor (hiPSC-CMs) and host (pigs) CMs. The corresponding secondary antibodies were: FITC conjugated donkey anti-mouse IgG, TRITC conjugated donkey anti-rabbit IgG, and Cy5 conjugated donkey anti-mouse IgM (all from Thermo Fisher, USA).

To determine the overall cell engraftment rate, real-time PCR for the human Y-chromosome copy number was performed in the hearts of CM and CM+Tb4 Groups (n = 8 for 4 weeks' follow-up and n = 6 for 12 weeks' follow-up) as described 4. Tissues of S3 - S5 of R2 and R4 and S2 and S5 of R3 were collected, digested overnight at 56 °C with proteinase K. The DNA was isolated from the digested buffer with a QIAGEN DNA isolation kit. The number of cells in each sample was determined by comparing the number of cycles to a standard curve calculated from the DNA of known quantities of hiPSC-CMs; then, the total number of cells in each heart was estimated from the sum of all samples from the same animal, and the engraftment rate was calculated as the number of cells in each animal divided by the number of viable cells administered (1.2 x 10^8^ x 80% = 9.6 x 10^7^). Analyses were performed with the SYBR Green kit (Fermentas, USA) on an Eppendorf Realplex PCR system (Eppendorf, USA) as previously described 29 with the following primers: sense-ATCAGCCTAGCCTGTCTTCAGCAA; anti-sense-TTCACGACCAACAGCACAGCAATG.

### Statistical analysis

Data was calculated and expressed as the mean ± standard deviation (SD). SPSS V25 was used for statistical analysis. To determine data distribution, P-P plot was used to test whether data was normally distributed or not. The difference between two groups was tested for significance using independent T-test. Comparisons among groups were analyzed for significance with one-way analysis of variance (ANOVA) with Tukey correction. For comparison of the incidence of ventricular arrhythmia, we divided each treatment group into positive and negative subgroups according to whether there was ventricular arrhythmia and weighted the cases. As the frequency was less than 40, we adopted Fisher's exact test. A value of p <0.05 was considered significant. The statistical analyses were performed with SPSS software (version 20).

## Results

### Purity of hiPSC-CMs

Flow-cytometry assessments of cTnT expression indicated that the purity of the hiPSC-CM population was 87.9 ± 5.4% upon completion of the differentiation protocol and increased to 98.7 ± 0.4% after 6 days of treatment with lactic acid (Figures S1A-B). Viability was assessed via trypan blue staining and indicated that 80.2 ± 2.2% of the hiPSC-CMs remained viable after recovery from liquid nitrogen (Figure S1C).

### Tb4 treatment modified the release of angiogenic paracrine factors from hiPSC-CMs

Treatment with Tb4 changed the production of angiogenic factors in cultured hiPSC-CMs (Tables S2 and S3). When evaluated using Human Angiogenesis Array, the abundance of angiopoietin-2, basic fibroblast growth factor, growth-regulated oncogene, fibroblast growth factor-4, inducible T cell alpha chemoattractant, monocyte chemotactic protein-4, tie-2, and VEGF was greater, while epithelial neutrophil-activating protein 78, insulin-like growth factor-1, matrix metalloproteinase-1, matrix metalloproteinase-9, platelet endothelial cell adhesion molecule, Tie-1, Urokinase receptor, VEGF receptor-2, and VEGF-D were less abundant, in the medium from Tb4-treated hiPSC-CMs than from hiPSC-CMs cultured without Tb4.

### Tb4 treatment protected hiPSC-CMs from hypoxic injury

**Figure 1:**
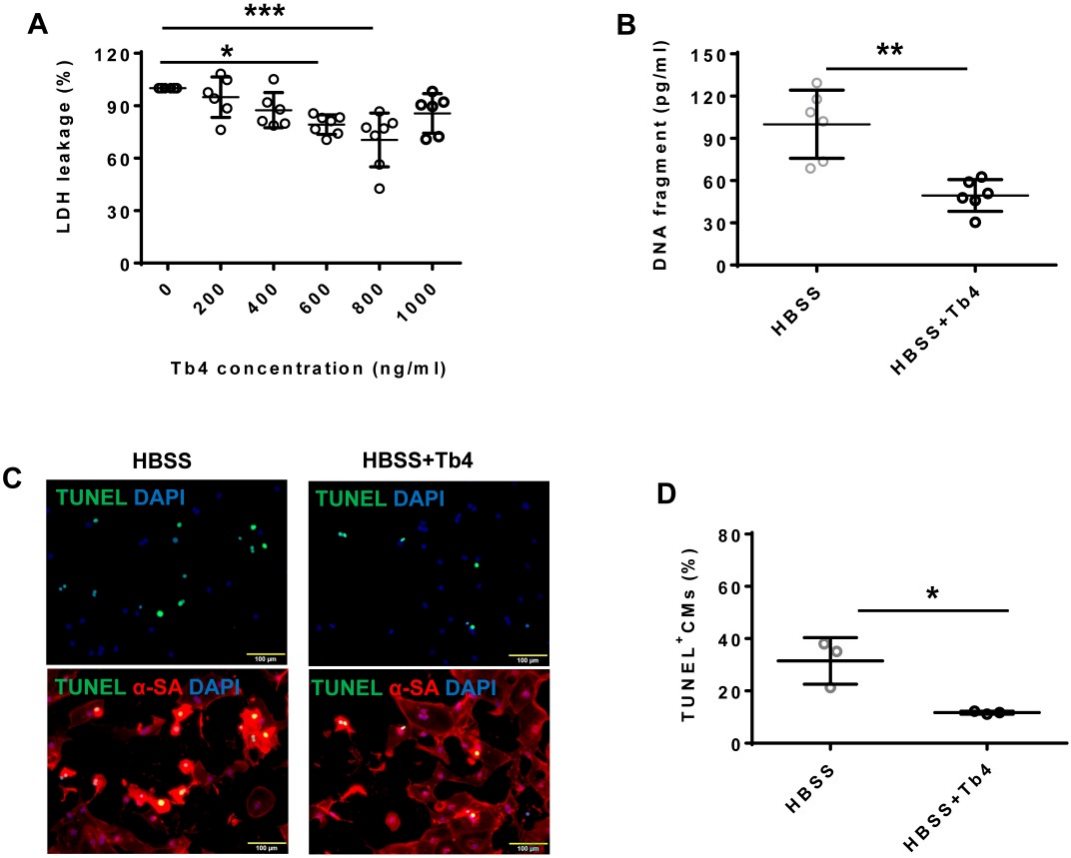

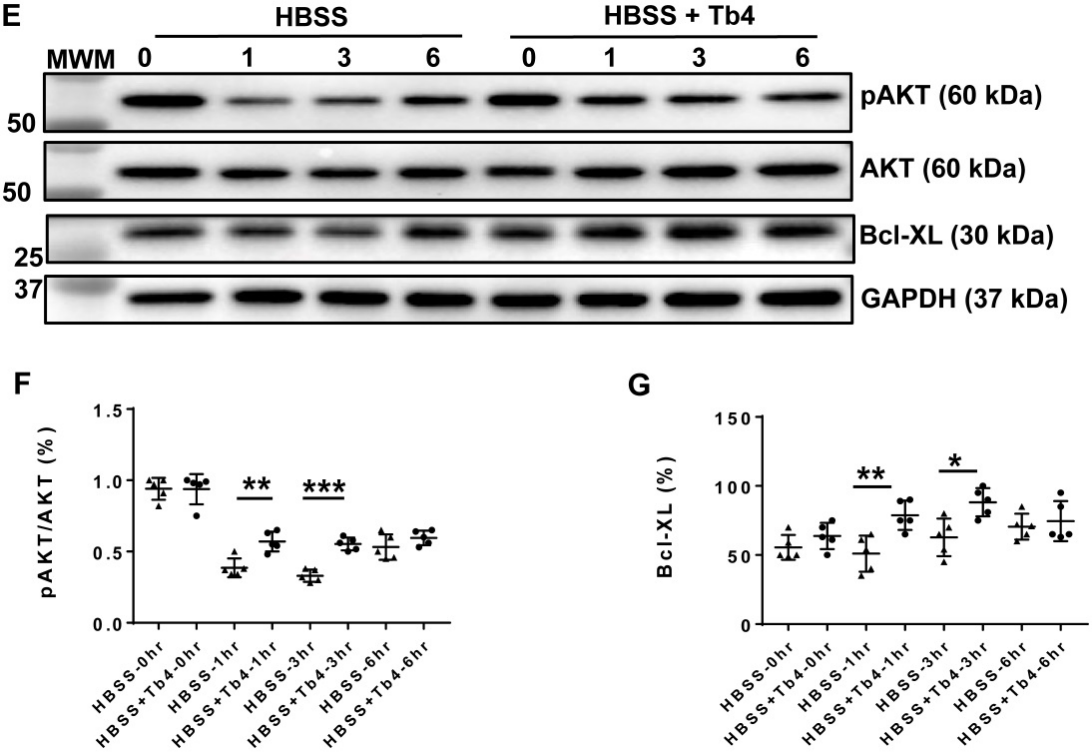
** Tb4 protects hiPSC-CM against hypoxic injury *in vitro*.** To determine cyto-protective effect of Tb4, 2x10^5^ hiPSC-CMs/well were cultured in 12-well plate. After washing thrice with DPBS, hiPSC-CMs were cultured in 500 μL Hanks balanced salt solution (HBSS) supplemented with or without Tb4 protein and cultured in an incubator with hypoxia condition for 24 h: 5% CO_2_, 94% N_2_, and 1% O_2._ **(A)** The concentrations of lactate dehydrogenase (LDH) in the cell culture medium were measured in the absence or presence of Tb4 and presented as a percentage of the measurements obtained in the absence of Tb4 (n = 6 for each concentration. One-way ANOVA analysis). **(B)** The concentration of DNA fragments in the cell culture medium were measured in the absence or presence of 600 ng/mL Tb4 (n = 6 for each sample. Independent T-test). **(C)** hiPSC-CMs cultured in HBSS medium supplemented without or with 600 ng/mL Tb4 were TUNEL-stained and counter-stained with DAPI after 24 hours' hypoxia. (α-SA: α-sarcomere actin. Bar = 100 μm). (**D**) hiPSC-CM apoptosis was quantified as the number of TUNEL^+^hiPSC-CMs over the total hiPSC-CMs per field (n = 3 for each sample. Independent T-test). **(E)** Representative Western Blot images of hiPSC-CMs cultured in HBSS medium supplemented without or with 600 ng/mL Tb4 for protein expressions of pAKT, AKT, and Bcl-XL. Protein expression level of GAPDH was used as an internal control. Cells were cultured under hypoxic condition and were harvested at 0, 1, 3, and 6 h after treatment. **(F)** Quantification of pAKT protein expressions, which were expressed as percentages of AKT protein levels after normalized with GAPDH protein. (n = 5 for each sample. Independent T-test). **(G)** Quantification of Bcl-XL protein expressions, which were presented as percentages of GAPDH protein levels. (n = 5 for each sample. Independent T-test). (*: p < 0.05; **: p < 0.01; ***: p < 0.001). Values are presented as the means ± SD.

Treatment with Tb4 (600 ng/mL) significantly reduced LDH leakage (Figure 1A), DNA damage (by 50%) (Figure 1B), and the proportion of TUNEL^+^cells (Figures 1C-D) when hiPSC-CMs were cultured under hypoxic conditions. Notably, hypoxia reduced AKT activity, while Tb4 significantly upregulated both AKT activity and Bcl-XL levels under hypoxic conditions (Figures 1E-G). Thus, Tb4 appeared to protect hiPSC-CMs from hypoxia-induced cellular damage by upregulating AKT activity and Bcl-XL protein expression.

### Tb4 promoted hiPSC-CM and hiPSC-EC proliferation *in vitro*

**Figure 2:**
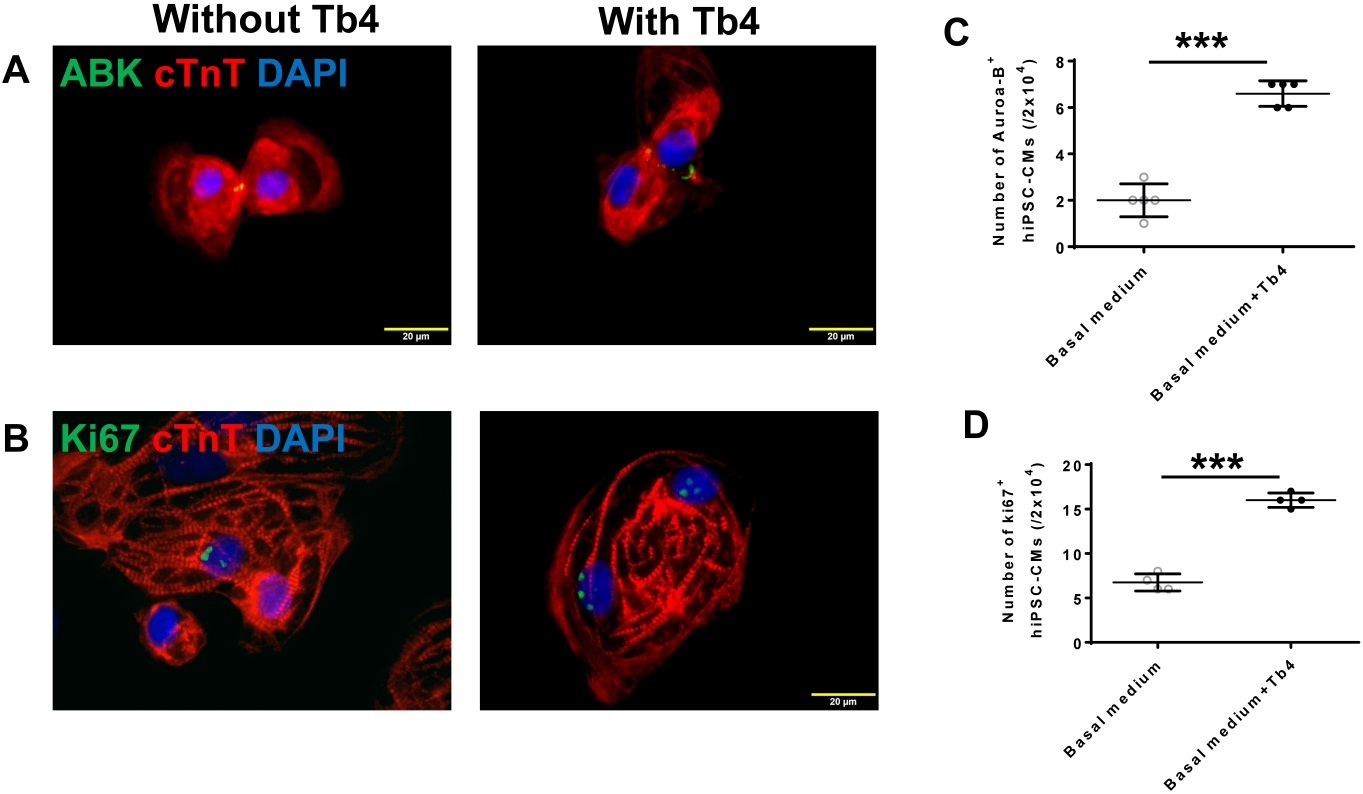
** Tb4 promoted hiPSC-CMs proliferation *in vitro*.** hiPSC-CM cultured in basal medium supplement with or without 600 ng/mL Tb4 for 4 days. Then cells were co-stained for expression of aurora-B kinase (ABK) and cardiac troponin T (cTnT) **(A)** or Ki67 and cTnT **(B)**. Quantification of hiPSC-CMs expressing ABK **(C)** or Ki67 **(D)** proteins. (Bar = 20 μm). (n = 5 or 4 for each staining. Independent T-test. ***: p < 0.001). Values are presented as the means ± SD.

Tb4 treatment significantly increased hiPSC-CM proliferation: expression of the cytokinesis marker ABK and the mitosis marker Ki67 was ~3-fold more common in Tb4-treated hiPSC-CMs than in hiPSC-CMs cultured without Tb4 (Figures 2A-D). Furthermore, when hiPSC-ECs were cultured with cEBM, or with Tb4 and fresh endothelial basal medium (Tb4+fEBM), or with Tb4 and cEBM (Tb4 + cEBM), proliferation measurements were significantly greater in the Tb4+fEBM treated cells than in cEBM-treated cells, and in Tb4 + cEBM-treated cells than in either of the other two treatment groups (Figures S2A-D). Analogous experiments conducted with human coronary artery SMCs cultured in cM231, Tb4 and fresh M231 (Tb4 + fM231), or Tb4 and cM321 (Tb4 + cM231) indicated that Tb4 also increased proliferation in cM231-cultured SMCs, while measurements in the cM231- and Tb4 + fM231-treated cells were similar (Figures S2E-H).

Notably, hiPSC-CMs continued to express high levels of cTnT and MLC2a, but only low levels of MLC2v and TNNI3, two weeks after Tb4 treatment, indicating that the hiPSC-CMs remained primarily atrial (Figures S2I&J).

### Tb4 released from gelatin microspheres

The gelatin microspheres were 15 to 50 μm in diameter (Figure S3A) and released Tb4 continuously for at least 14 days *in vitro* (Figure S3B). *In vivo*, Tb4-his concentration in rat serum was 28.9 ng/mL on day-2 and was almost negligible on day 14 after transplantation (Figure S3C). The remaining Tb4-his was 34.8 ± 5.3 ng in a rat left ventricular anterior wall on day-14 after transplantation. When hiPSC-CMs, hiPSC-ECs, and SMCs were cultured in their respective growth media with Tb4-microspheres, the morphology of hiPSC-CMs and hiPSC-ECs was nearly unchanged over 14 days, while SMCs proliferated to reach nearly 100% confluence in just 4 days. Measurements of LDH leakage did not change significantly in any of the three cell types throughout the culture periods (Figure S4).

### Co-treatment with hiPSC-CMs and Tb4-microspheres improved recovery of cardiac function and reduced infarct size in pigs after MI and treatment

Because Tb4 protected hiPSC-CMs from hypoxia *in vitro*, we investigated whether Tb4 could enhance hiPSC-CM viability and the potency of transplanted hiPSC-CMs for cardiac repair in infarcted pig hearts. MI was surgically induced by permanently ligating the coronary artery, and animals were treated with basal RPMI medium (the MI Group), with medium containing Tb4-microspheres (the Tb4 Group), with medium containing hiPSC-CMs (the CM Group), or with medium containing both the Tb4-microspheres and hiPSC-CMs (the Tb4 + CM Group); a fifth group of animals underwent sham surgery and recovered without any of the experimental treatments (the Sham Group) (Table S1).

**Figure 3:**
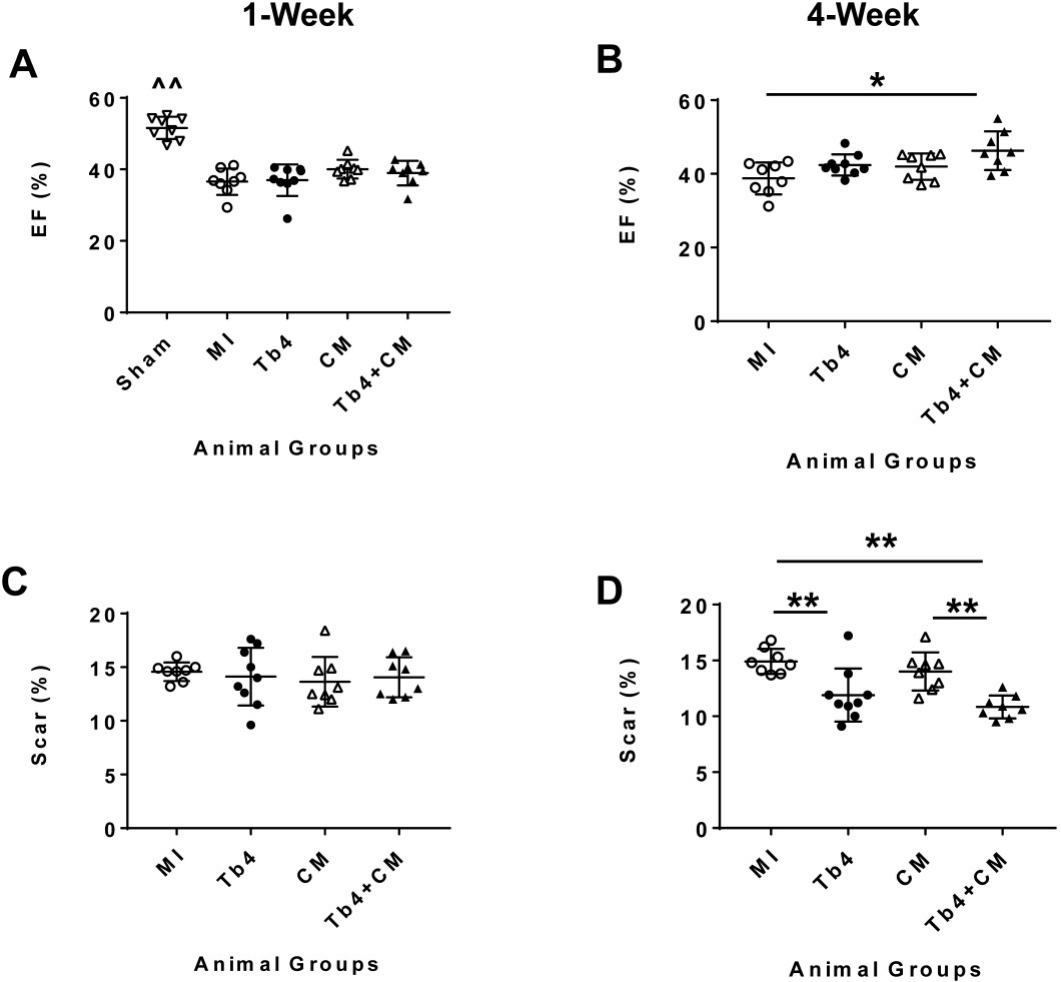
** Tb4 increased reparability potency of hiPSC-CM in improving systolic function and reduced scar area in the pig hearts at week 4 after MI and treatment. L**eft ventricular ejection fraction (LVEF) of the pig hearts as assessed by cardiac MRI at weeks-1 **(A)** and -4 **(B)** after MI and treatment. The infarct area measured by cardiac MRI at weeks-1 **(C)** and -4 **(D)** after MI and treatment. (n = 8 - 9 for each group. One-way ANOVA. ^^: vs any other group, *: p < 0.05, and **: p < 0.01). Values are presented as means ± SD.

One week after MI induction and treatment, MRI assessments of LV ejection fraction (LVEF) in the four groups that underwent MI did not differ significantly, but were significantly lower than in the Sham Group (p < 0.01) (Figure 3A and Figure S5). At week 4, LVEFs in the MI and CM Groups remained similar and were largely unchanged from week 1, while LVEFs in both the Tb4 and Tb4 + CM Groups increased significantly between the two time points (p < 0.01); however, only measurements in the Tb4 + CM Group were significantly greater than those in the MI Group (p < 0.05) (Figure 3B). Change in LVEF (∆LVEF) was also significantly greater in the Tb4 + CM Group than either the MI or CM Groups (Figure S6A).

Scar areas were similar at week 1 among the four groups that underwent MI (Figure 3C) and did not change significantly between weeks -1 and -4 in the MI and CM Groups. Measurements in the Tb4 Group tended to decline between the two time points; however, significant improvement from week 1 to week 4 was only observed in the Tb4 + CM Group (p < 0.01). Scar areas at week 4 were also significantly smaller in the Tb4 Group than the MI Group (p < 0.01), and in the Tb4 + CM Group than in either the MI or CM Group (p < 0.01) (Figure 3D), while the change in scar area (∆scar) was significantly greater in the Tb4 + CM Group than in either the MI or CM Group (p < 0.05) (Figure S6B). Collectively, these observations indicate that co-treatment with Tb4 and hiPSC-CMs, but not with hiPSC-CMs alone, significantly improved both cardiac performance and scar size in infarcted pig hearts.

### Co-treatment with hiPSC-CMs and Tb4-microspheres increased wall thickness at the infarct site

Transmural scar was observed in Masson-trichrome-stained sections from the hearts of animals in all groups that underwent MI induction (Figures S7A-E), and wall thickness in the infarcted region was significantly lower (p < 0.01) in MI, Tb4, CM, and Tb4 + CM animals than in the Sham Group at week 4 (Figure S7F). However, islands of porcine CMs were observed in the scared area of the hearts from animals in the Tb4 and Tb4 + CM Groups as they were not immunostained positively for human nuclei. The infarcted wall was significantly thicker (p < 0.05), by ~20%, in Tb4 + CM-treated animals than in the MI Group (p < 0.05).

### Co-treatment with hiPSC-CMs and Tb4 microspheres improved vasculogenesis after MI

**Figure 4:**
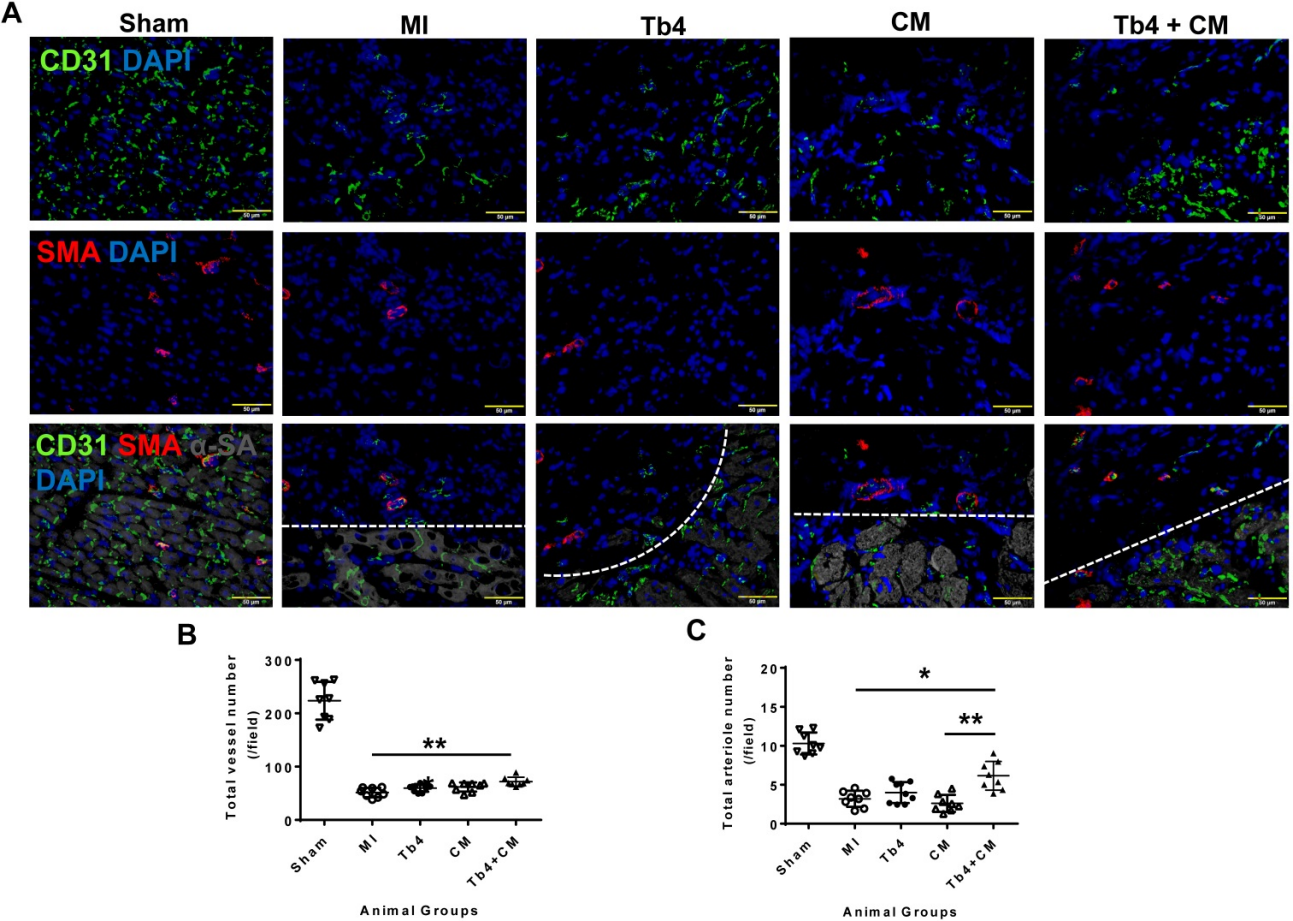
** Co-treatment with Tb4 and hiPSC-CM enhanced vasculogenesis in pig hearts at weeks 4 after MI and treatment. (A)** Representative images of fluorescence immunostaining for CD31, smooth muscle actin (SMA), and α-sarcomere actin (α-SA) in pig hearts from the Sham, MI, Tb4, CM, and Tb4 + CM Groups. Images of the MI, Tb4, CM, and Tb4 + CM Groups were taken at the border zone of infarct. Dash lines distinguish the infarct zone (above or on left-side of the line) from the survival CMs (below or on the right-side of the line). **(B)** Total vessel number per field (40 x magnification) was determined by counting CD31^+^ vascular numbers. **(C)** Total arteriole number per field (40 x magnification) was determined by counting vessels co-expressed CD31 and SMA. (Bar = 50 µm). (n = 8 or 9 for each group, One-way ANOVA: *: p < 0.05; **: p < 0.01; ***: p < 0.001). Values are presented as means ± SD.

To determine whether co-treatment with hiPSC-CMs and Tb4 increased vascular and arteriole densities, cryosections of heart tissue were stained for expression of the EC-marker CD31 and SMA (Figure 4A). In the border zone of the infarct at week 4, total (CD31^+^) vessel density and arteriole (SMA^+^) density were significantly lower in animals from all infarcted animal groups than in the Sham Group, but both measurements were significantly greater in the Tb4 + CM Group than in the MI Group (total vessel density: p < 0.001; arteriole density: p < 0.05), and arteriole density was greater in Tb4 + CM animals than in CM animals (p < 0.01). Most of the border-zone capillaries were located in regions with surviving cardiomyocytes. Thus, co-treatment with hiPSC-CMs and Tb4 enhanced both the angiogenic and arteriogenic response to MI.

### Tb4 microspheres improved the engraftment of transplanted hiPSC-CMs

**Figure 5:**
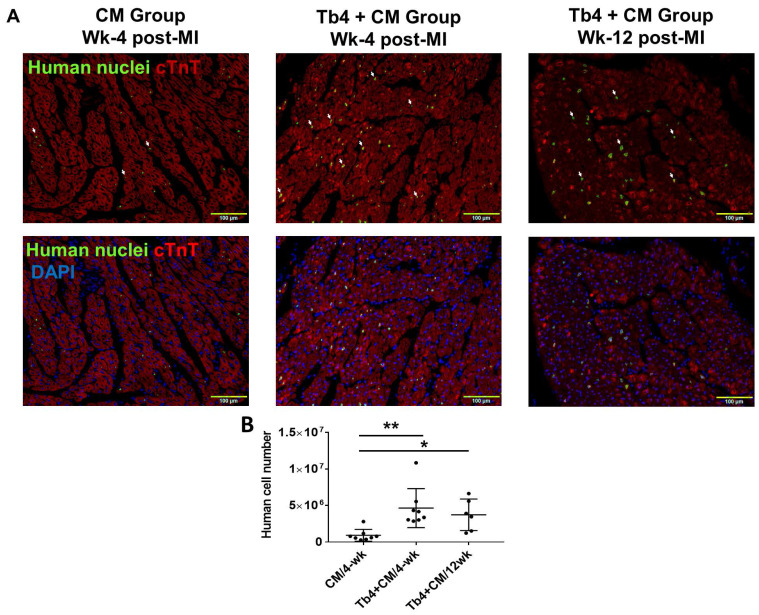

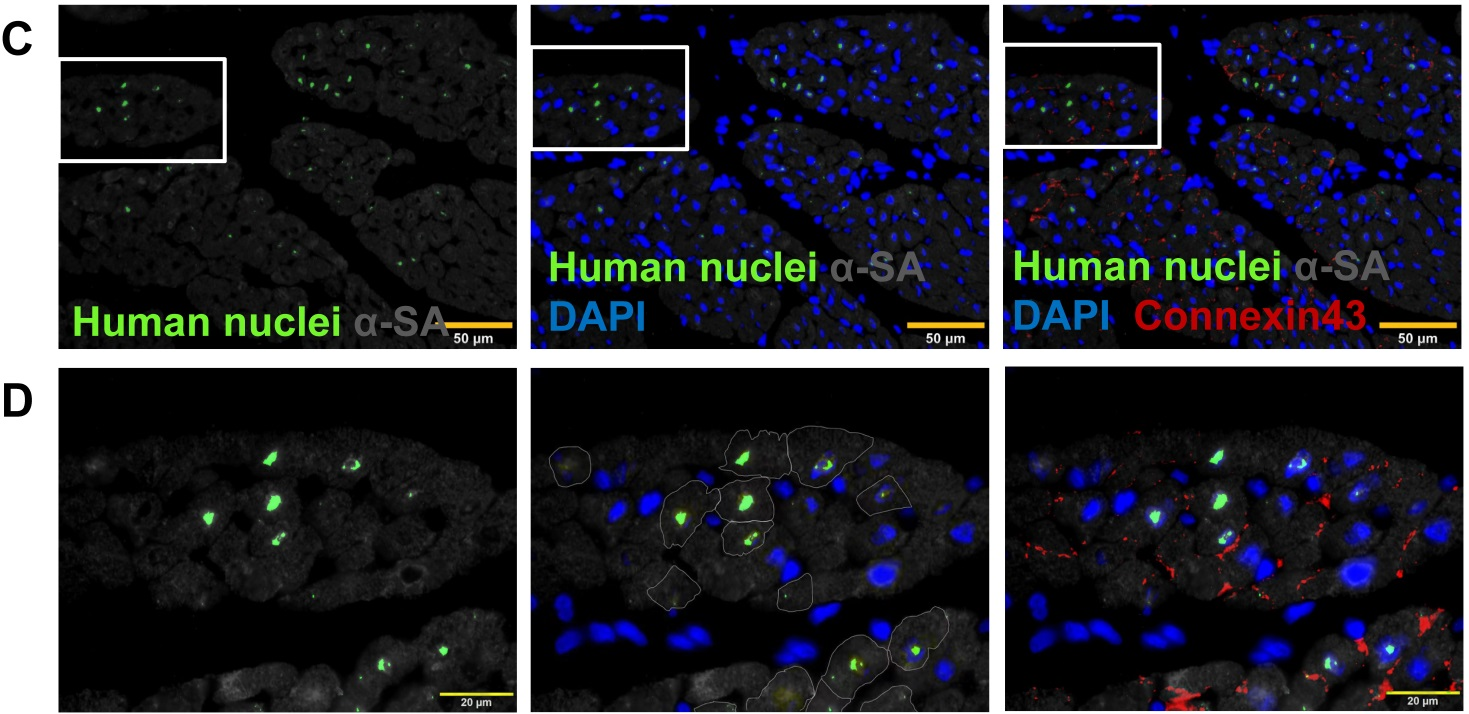
** Co-treatment with Tb4 enhanced hiPSC-CM engraftment in porcine hearts after MI and treatment. (A)** Representative images of fluorescence immunostaining for human nuclei and cardiac troponin T (cTnT) expressions in pig hearts of the CM Group at weeks -4 and the Tb4 + CM Group at weeks -4 and -12 after MI and treatment. (Bar = 100 μm. White arrows indicate representative CMs expressing human nuclei antigen). **(B)** Quantification of human cell engraftments in pig hearts of the CM and Tb4 + CM Groups. **(C)** Connexin43 protein expression between implanted hiPSC-CMs and host pig CMs (Bar = 50 μm). **(D)** The selected areas from C were magnified. (Bar = 20 μm). Lines were drawn to define the morphology of engrafted hiPSC-CMs. (n = 8 or 6 for each group, One-way ANOVA: *: p < 0.05; **: p < 0.01). Values are presented as the means ± SD.

Because the transplanted hiPSC-CMs were of human origin, engrafted cells were identified in the hearts of CM and Tb4 + CM animals by staining sections of myocardial tissue with antibodies specific for human nuclei (Figure 5A). When visualized with fluorescent secondary antibodies, the engrafted hiPSC-CMs had integrated with the native porcine CMs and formed organized myocardial structures. Furthermore, since the hiPSC-CMs were genetically male, the engraftment rate was estimated via quantitative PCR measurements of the human Y chromosome: engraftment was significantly greater at both week-4 (4.8%) and week-12 (3.9%) in Tb4 + CM treated hearts than in hearts from the CM group at week-4 (0.96%) (Figure 5B). Notably, immunofluorescence analyses also found evidence of Connexin-43 protein expression between engrafted hiPSC-CMs and native porcine CMs (Figures 5C&D).

### Tb4-microspheres promoted cardiac cell proliferation *in vivo*

**Figure 6:**
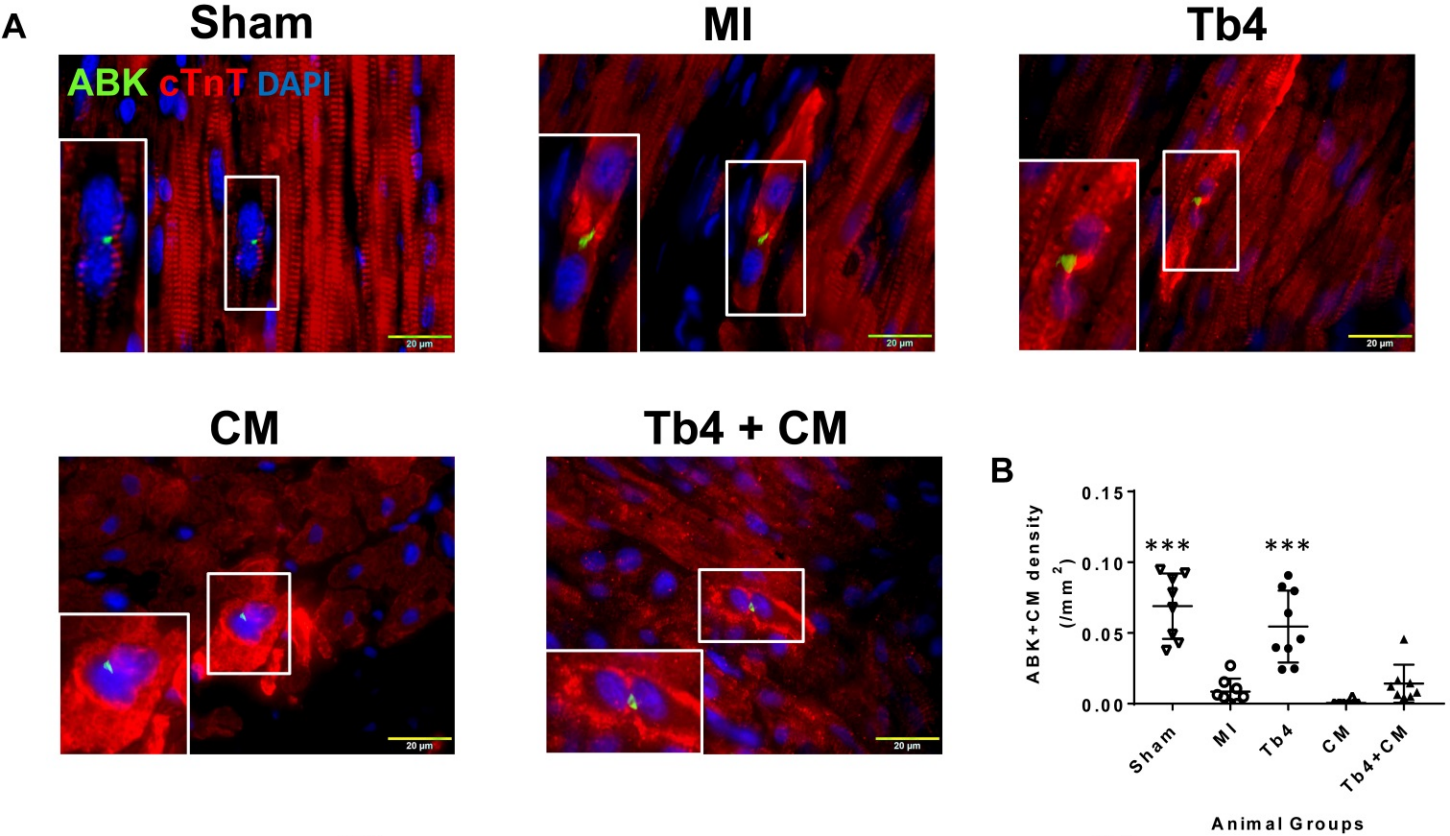

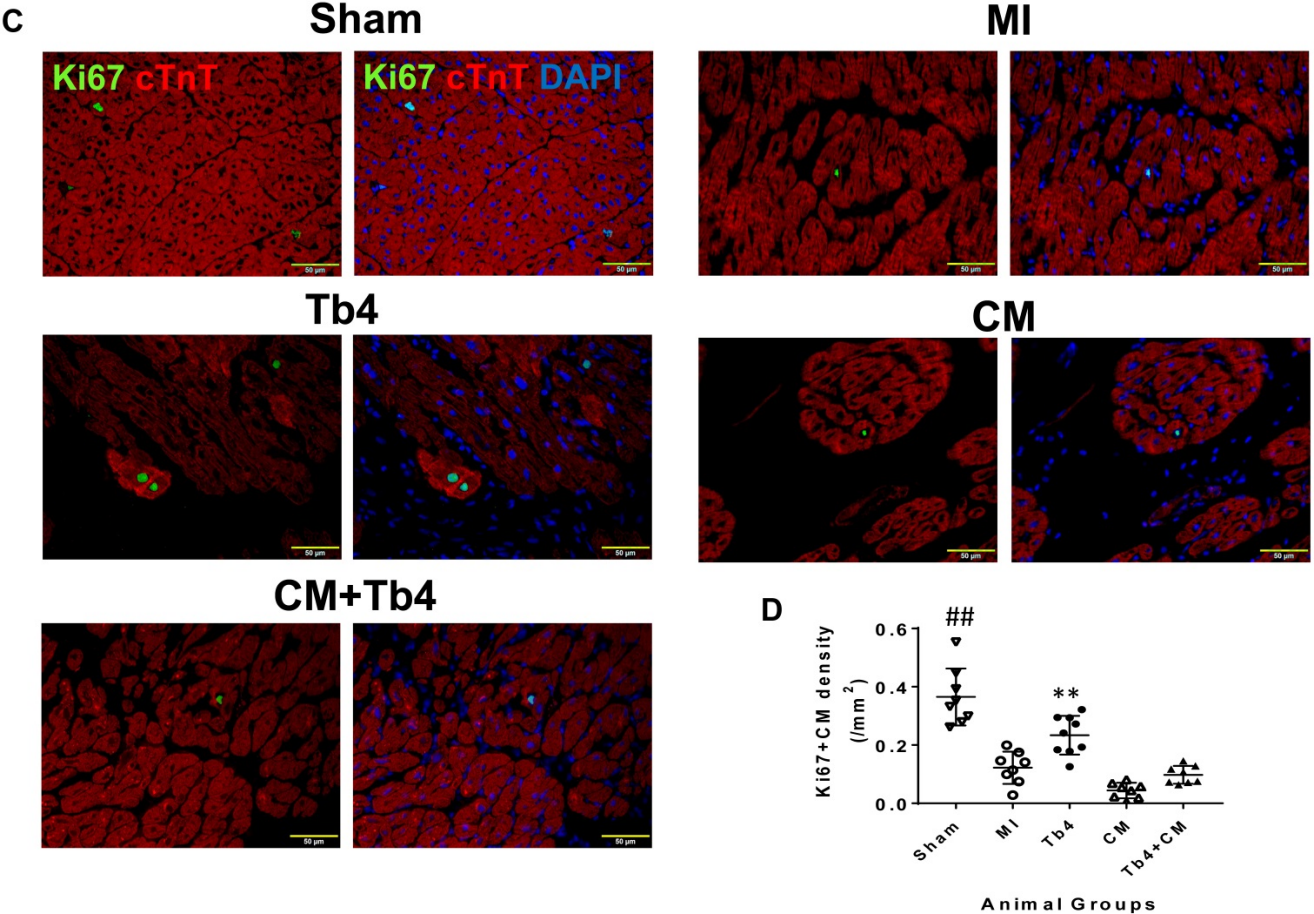
** Cardiomyocyte proliferation in pig hearts after MI and treatment. (A)**. Representative images of CM undergoing cytokinesis: Aurora B kinase (ABK) expressing CMs with disassembled sarcomeres and cleavage furrows in the Sham, MI, Tb4, CM**,** and Tb4 + CM Groups at Week-4 post-MI and treatment. The selected areas were shown, respectively, at high magnification. (Bar = 20μm). **(B)** Quantitative analysis of the number of ABK^+^CMs in all animal groups. (n = 8 or 9 for each group. One-way ANOVA: **: P < 0.01 vs MI, CM, and Tb4 + CM Groups). **(C)**. Representative images of Ki67 expressing CMs in the Sham, MI, Tb4, CM**,** and Tb4 + CM Groups at Week-4 after MI and treatment. (Bar = 50 μm). **(D)** Quantitative analysis of the number of Ki67^+^CMs in all animal groups. (n = 8 or 9 for each group. One-way ANOVA. **: P < 0.01 vs the MI, CM, and Tb4 + CM Groups; ***: P < 0.001 vs the MI, CM, and Tb4 + CM Groups; ##: P < 0.01 vs every other group). Values are presented as the means ± SD.

Sections stained for ABK and cardiac troponin T (cTnT) (Figure 6A) indicated that MI dramatically reduced CM cytokinesis in pig hearts: the CM cytokinesis rate was 0.069 ± 0.02 cells/mm^2^ in hearts from the Sham Group, compared to just 0.009 ± 0.009 cells/mm^2^ in MI animals (p < 0.001), and just 6 of 8 animals in the MI Group displayed any evidence of CM cytokinesis. Treatment with Tb4 after MI partially restored CM cytokinesis (0.055 ± 0.03 cells/mm^2^, p < 0.001 versus MI). However, dividing CMs were identified in only one animal from the CM Group, and the rate of CM cytokinesis was even lower in the CM Group (0.0006 ± 0.0016 cells/mm^2^) than in MI animals, though not significantly. Measurements in Tb4 + CM animals (0.014 ± 0.013/mm^2^) were somewhat higher than the CM Group, but not significantly, and remained significantly lower than in the Sham or Tb4 Group (Figure 6B). Furthermore, although Ki67^+^CMs were identified in the hearts of all animals from all experimental groups (Figure 6C), they were most common in the hearts from Sham animals (p < 0.01 versus every other group) and significantly more prevalent in the Tb4 Group than in the MI, CM, or Tb4+CM Group (p < 0.01) (Figure 6D). Ki67^+^CMs were somewhat more common in Tb4 + CM treated than in CM-treated animals, but not significantly.

Notably, Ki67^+^ECs were significantly more common in the hearts from the Tb4 (p < 0.01) and TB4 + CM (p < 0.05) treated animals than from Sham, MI, or CM animals (Figures S8A&B). Although Ki67^+^SMCs were most common in Sham animals (p < 0.01 versus every other group), they were significantly more prevalent in Tb4 + CM treated hearts than in the hearts from the MI and CM Groups (p < 0.01), and in the hearts from the Tb4 Group than from the CM Group (p < 0.05) (Figures S8C&D).

Cells expressing the immune marker CD11b were significantly more common in the hearts of animals from all four groups that underwent MI than in the hearts from the Sham Group (p < 0.001 versus the MI and CM Groups, p < 0.01 versus the Tb4 and Tb4 + CM Groups) (Figures S9A-F), but the greatest CD11b^+^cell density was observed in MI animals (p < 0.001 versus every other group), while measurements in the Tb4, CM, and Tb4+CM Groups did not differ significantly. Most of the CD11b^+^cells were located in the scarred region of infarcted hearts and in the epicardium of animals in the Sham Group.

### hiPSC-CM transplantation did not increase ventricular arrhythmia

**Figure 7:**
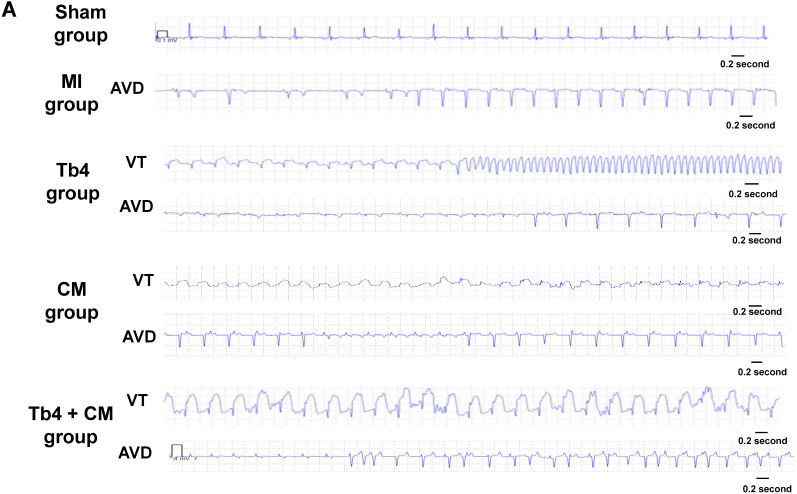

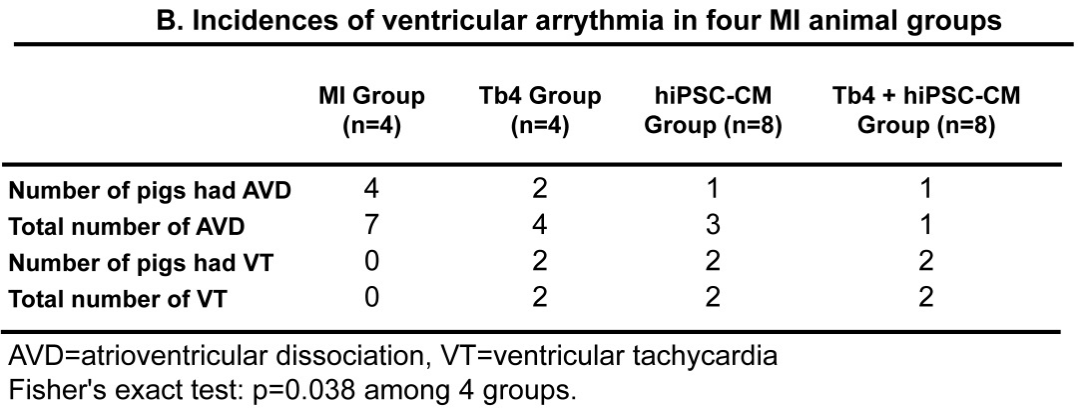
** hiPSC-CM transplantation did not increase the incidence of ventricular arrythmia after MI and treatment. (A)** Representative normal electrocardiogram images in the Sham Group and image of atrioventricular dissociation (AVD) or ventricular tachycardia (VT) in the MI, Tb4, CM, and Tb4 + CM Groups after MI and treatment. **(B)** Quantification of incidences of VT in the MI, Tb4, CM, and Tb4 + CM Groups. (n = 4 - 8 for each group).

The results from previous studies in both monkeys and pigs suggest that hESC-CM transplantation can promote arrhythmogenesis 9, 20; thus, ECGs were continuously recorded in animals from this study with implanted loop recorders. Ventricular arrhythmia was not detected in animals from the Sham Group, and no animal in any of the groups that underwent MI induction developed spontaneous ventricular fibrillation (Figures 7A&B) through 4 weeks. Within 48 h of MI induction, AVD was observed in 4 animals from the MI Group, AVD and VT each occurred in 2 animals from the Tb4 group, AVD and VT were each observed in 1 animal from the CM Group, and AVD and VT was found only in 1 and 2 pigs, respectively, in the Tb4 + CM Group. One VT also occurred in a third animal from the CM group at week 3. Statistical analysis identified significant variation across all four groups that underwent MI induction (p = 0.038, Fisher's exact test), but pairwise comparisons did not differ significantly between any two groups.

### hiPSC-CM transplantation did not induce tumor formation in major organs

Tumorigenesis was evaluated in six pigs from the Tb4 + CM Group using MRI at 1 (Videos S1-S4) and 12 (Videos S5-S10) weeks after MI induction and treatment. One animal died during the MRI scanning procedure at week-4. When images obtained at both time points in the same animal were compared, no evidence of lesion formation was observed in any animal**.**

## Discussion

Although large numbers of hESC-CMs have been delivered to the infarcted hearts of non-human primates and pigs 8, 20, no previous study has explored the efficacy and safety of clinically relevant doses of hiPSC-CMs in large animal models of acute myocardial injury. We addressed this gap in the development of hiPSC-CMs for cellular cardiomyoplasty by transplanting 1.2
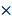
10^8^ hiPSC-CMs into the hearts of pigs after MI. Furthermore, since Tb4 is known to enhance the survival and migration of cardiac cells 11, to promote cardioprotection, angiogenesis, and vasculogenesis 15, 16, and to improve cell engraftment 18; we also investigated whether the controlled release of Tb4 could improve the reparative potency of hiPSC-CMs. Co-treatment with Tb4-microspheres enhanced hiPSC-CM viability and engraftment and induced vasculogenesis and the proliferation of CMs and ECs, which inhibited scar expansion and improved LV systolic function without increasing the incidence of ventricular arrhythmia. We also found no evidence of tumor formation in major organs of immunosuppressed animals.

Cardiac MRI data indicated that scar size declined at week-4 after MI and treatment in both the Tb4 and Tb4 + CM Groups, but only Tb4 + CM animals displayed significant improvements in LVEF, vascular density, and arterial density. Thus, co-treatment with Tb4-microspheres and hiPSC-CMs, but neither treatment alone, led to robust improvements in myocardial repair. hiPSC-CM engraftment was also greater in Tb4 + CM than in CM animals, which suggests that Tb4 had a cytoprotective effect on the transplanted cells, and the improvements in EF and scar size observed in Tb4 + CM animals at week-4 and were maintained at week-12.

*In vitro* studies showed that Tb4 protected hiPSC-CMs from hypoxia by upregulating AKT activity and Bcl-XL expression, which is consistent with our previous studies showing that Tb4 significantly reduced apoptosis in hypoxia-cultured swine mesenchymal stem cells by increasing the expression of Bcl-XL and reducing caspace-8 activation 18. These observations are likely linked, because Tb4 can activate integrin linked kinase to activate AKT, which subsequently upregulates transcription factors that increase the expression of caspase inhibitors, such as Bcl-XL, to promote cell survival. Bcl-XL is believed to disrupt formation of the death-inducing signal complex, which is required for caspace-8 activation 30, and may impede the apoptotic response to declines in mitochondrial membrane potential by maintaining mitochondrial integrity and limiting cytochrome c release 31. hESC-CMs have also been treated with a pro-survival cocktail before administration 6, but the prolonged release of Tb4 from gelatin microspheres may be a more clinically relevant method for improving cell survival, because the pro-survival cocktail included Matrigel, which is not suitable for clinical applications. Tb4 also modified the production of angiogenic factors in hiPSC-CMs: the expression of basic fibroblast growth factor, fibroblast growth factor-4, and VEGF was greater in Tb4-treated hiPSC-CMs than in the absence of Tb4, which likely also contributed to the observed improvements in cytoprotection and vasculogenesis observed in Tb4 + CM hearts 29, 32, 33, because the first 2 factors are known to induce arteriogenesis 34, 35.

Tb4 transplantation also induced both EC and CM proliferation *in vivo*, which may have important implications for future studies, because MI inhibited CM cytokinesis in native porcine CMs, and CM cytokinesis was further suppressed in animals treated with hiPSC-CMs alone. Notably, persistent AKT activation increased mitosis and cytokinesis in both neonatal and adult CMs 36, which suggests that if the release of Tb4 from gelatin microspheres could be even more prolonged (e.g., for several months) cardiac regeneration may be improved via the induction of cytokinesis in native porcine CMs.

Cyclosporin was administered for immune suppression. Nevertheless, immune cell infiltration was still observed in all 4 groups that underwent MI induction and was most severe in the MI Group; thus, cyclosporin alone may not sufficiently suppress the immune response. A combination of different immunosuppressive drugs, including methylprednisolone, Abatacept, and cyclosporin 9, may be more effective, but this (or another) combination of immunosuppressive drugs must be optimized to minimize side-effects before clinical translation.

Loop recorder data showed that hiPSC-CM transplantation did not increase the incidence of ventricular arrhythmia, including AVD or VT; in fact, hiPSC-CM implantation tended to reduce ventricular arrhythmia (but not significantly). This result is consistent with our previous study, which found no increase in ventricular arrhythmias (induced by programmed electrical stimulation) among animals treated with hiPSC-CMs or hiPSC-CMs and vascular cells 4, but contradicts the results from previous investigations with hESC-CMs and miPSC-CMs 8, 20, which indicated that the cells significantly increased ventricular tachycardia or fibrillation when administered to the hearts of non-human primates and pigs. These discrepancies may be partially attributable to differences in the animal model: the endogenous rates of contraction in porcine CMs and hiPSC-CMs are similar, but the non-human primate studies were conducted in macaques, whose a mean heart rate (130 beats/min) is considerably faster than the rate in human hearts 37 and, consequently, may not be compatible with the rate of contraction in hESC or hiPSC-CMs. Furthermore, if the implanted cells formed isolated islands that failed to integrate with native CMs, the donor cells would not contract synchronously with the rest of the myocardium. Here, we found that the transplanted cells formed gap junctions with host CMs, suggesting that the hiPSC-CMs may be electromechanically coupled to the host cells.

Another safety concern associated with pluripotent stem cell therapy is the potential for tumor formation 38; however, when unpurified hiPSC-CMs (88% of which were CMs) were collected 7 days after contractions were first observed (i.e., 15-17 days after differentiation was initiated) and transplanted into the hearts of immunosuppressed pigs, whole body MRI found no evidence of tumor lesion formation in any of the 6 surviving animals 12 weeks after cell administration. Collectively, these observations suggest that hiPSC-CM transplantation does not increase the risk of arrhythmogenesis or tumorigenesis.

In conclusion, treatment combining the controlled release of Tb4 from gelatin microvesicles and hiPSC-CM transplantation was safe and more efficacious for myocardial repair than treatment with hiPSC-CMs alone when evaluated in porcine model of myocardial injury. Tb4 enhanced hiPSC-CM engraftment and induced vasculogenesis and the proliferation of CMs and ECs, which improved measures of LV function and scar size without increasing the risk of ventricular arrhythmia in infarcted pig hearts or of tumorigenesis in the major organs of immunosuppressed pigs. This co-treatment between prolonged Tb4 release and hiPSC-CM implantation has important clinical implications for the treatment of MI; for example, the treatment could be delivered via a catheter during percutaneous transluminal coronary angioplasty or by direct intramyocardial injection as adjunctive therapy during coronary artery bypass grafting surgery.

## Supplementary Material

Supplementary figures, tables, video legends.

Supplementary video 1.

Supplementary video 2.

Supplementary video 3.

Supplementary video 4.

Supplementary video 5.

Supplementary video 6.

Supplementary video 7.

Supplementary video 8.

Supplementary video 9.

Supplementary video 10.

## Abbreviations

ABK: aurora B kinase
α-SA: α-sarcomere actin
AVD: atrioventricular dissociation
CM: cardiomyocyte
cTnT: cardiac troponin T
DAPI: 4′,6-diamidino-2-phenylindole
DPBS: Dulbecco's phosphate-buffered saline
EBM: Endothelial basal medium
ECG: electrocardiogram
ECs: endothelial cells
EF: ejection fraction
EGM: Endothelial growth medium
GAPDH: glyceraldehyde phosphate dehydrogenase
GM: gelatin microspheres
HBSS: Hanks balanced salt solution
hESC: human embryonic stem cell
hESC-CM: human embryonic stem cell derived cardiomyocyte
hPSC: human pluripotent stem cell
hiPSC-CM: human induced pluripotent stem cell derived cardiomyocyte
hiPSC-ECs: human induced pluripotent stem cell derived endothelial cells
LDH: lactate dehydrogenase
LV: left ventricle
M231: Medium 231
MI: myocardial infarction
MLC2a: atrial myosin light chain 2
MLC2v: ventricular myosin light chain 2
miPSC-CM: monkey induced pluripotent stem cells derived CM
MRI: magnetic resonance imaging
RPMI-: RPMI medium without glucose
SMA: smooth muscle actin
SMC: smooth muscle cells
Tb4: thymosin β4
TRITC: Tetramethylrhodamine
TUNEL: Terminal deoxynucleotidyl transferase dUTP Nick-End Labeling
VEGF: vascular endothelial growth factor
VT: ventricular tachycardia
