# Supplementary material for: Thymosin β4 increases cardiac cell proliferation, cell engraftment, and the reparative potency of human induced-pluripotent stem cell-derived cardiomyocytes in a porcine model of acute myocardial infarction
Source: Theranostics. 2021 Jun 26;11(16):7879–95. doi: 10.7150/thno.56757 (PMC8315077; doi:10.7150/thno.56757)
Supplement: Supplementary file 1 — Supplementary figures, tables, video legends. [file thnov11p7879s1.pdf]

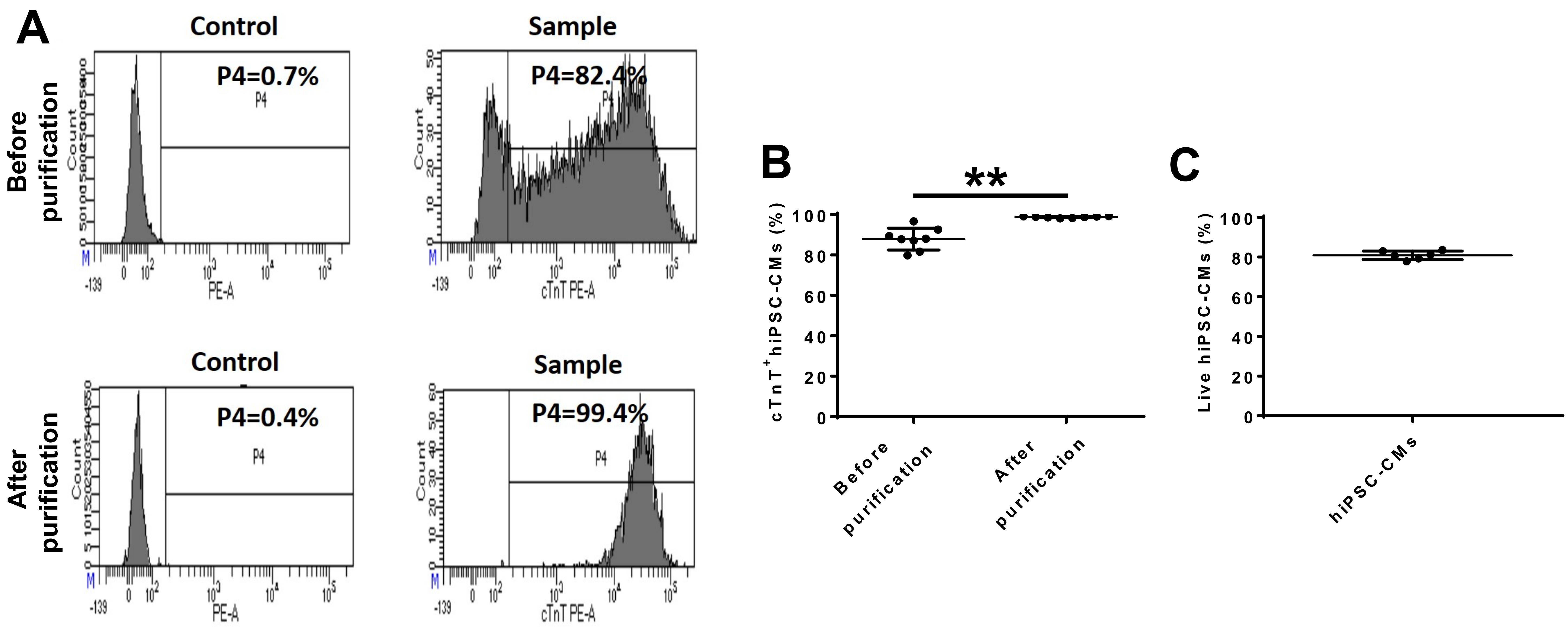

Figure S1: Purity and viability of hiPSC-CMs. (A) Representative flow cytometry images of cTnT⁺hiPSC-CMs before and after purification. **(B)** Quantification of cTnT⁺hiPSC-CM before and after purification. (n = 8) (Independent T-test. **: $p < 0.01$). **(C)** Percentage of viable hiPSC-CMs after recovered from liquid nitrogen. (n = 6). Values are presented as the means ± SD.

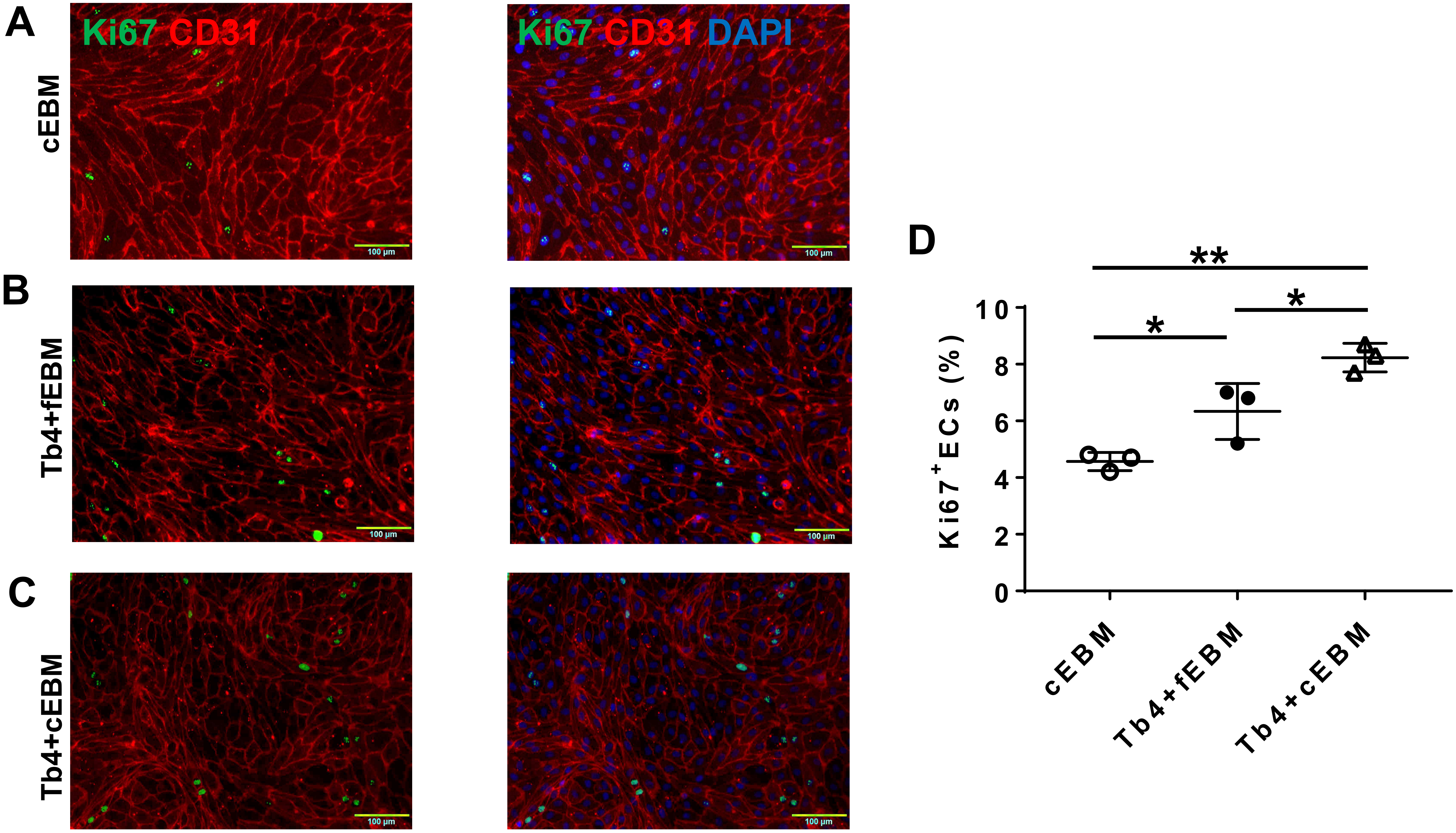

Figure S2. Representative images of hiPSC-ECs cultured in either hiPSC-CM conditioned endothelial basal medium (cEBM) **(A)**, or fresh endothelial basal medium (fEBM) supplemented with 600 ng / mL Tb4 **(B)**, or cEBM supplemented with 600 ng / mL Tb4 **(C)** for 48 h, and then cells were co-stained for expression of Ki67 and CD31 proteins. **(D)** Quantification of hiPSC-ECs expressing Ki67. (Bar = 100 μm). (n = 3. Independent T-test. *: $p < 0.05$, **: $p < 0.01$). Values are presented as the means ± SD.

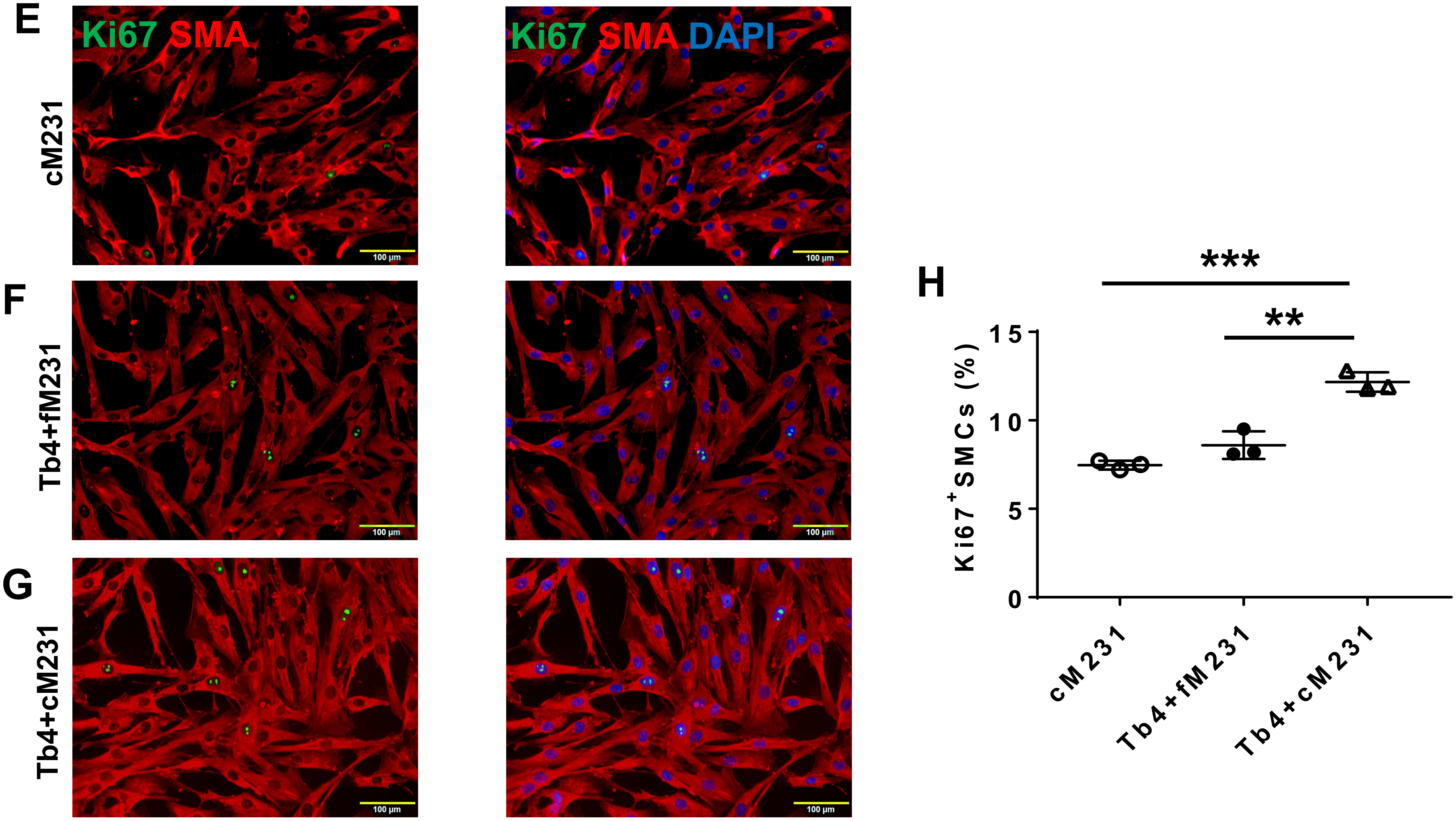

Figure S2. Representative images of smooth muscle cells (SMCs) cultured in either hiPSC-CM conditioned M231 medium (cM231) **(E)**, or fresh M231 medium (fM231) supplemented with 600 ng / mL Tb4 **(F)**, or cM231 supplemented with 600 ng / mL **(G)** for 24 h, and then cells were co-stained for expression of Ki67 and smooth muscle actin (SMA) proteins. **(H)** Quantification of SMCs expressing Ki67. (Bar = 100 µm). (n = 3. Independent T-test. **: $p < 0.01$, ***: $P < 0.001$). Values are presented as the means ± SD.

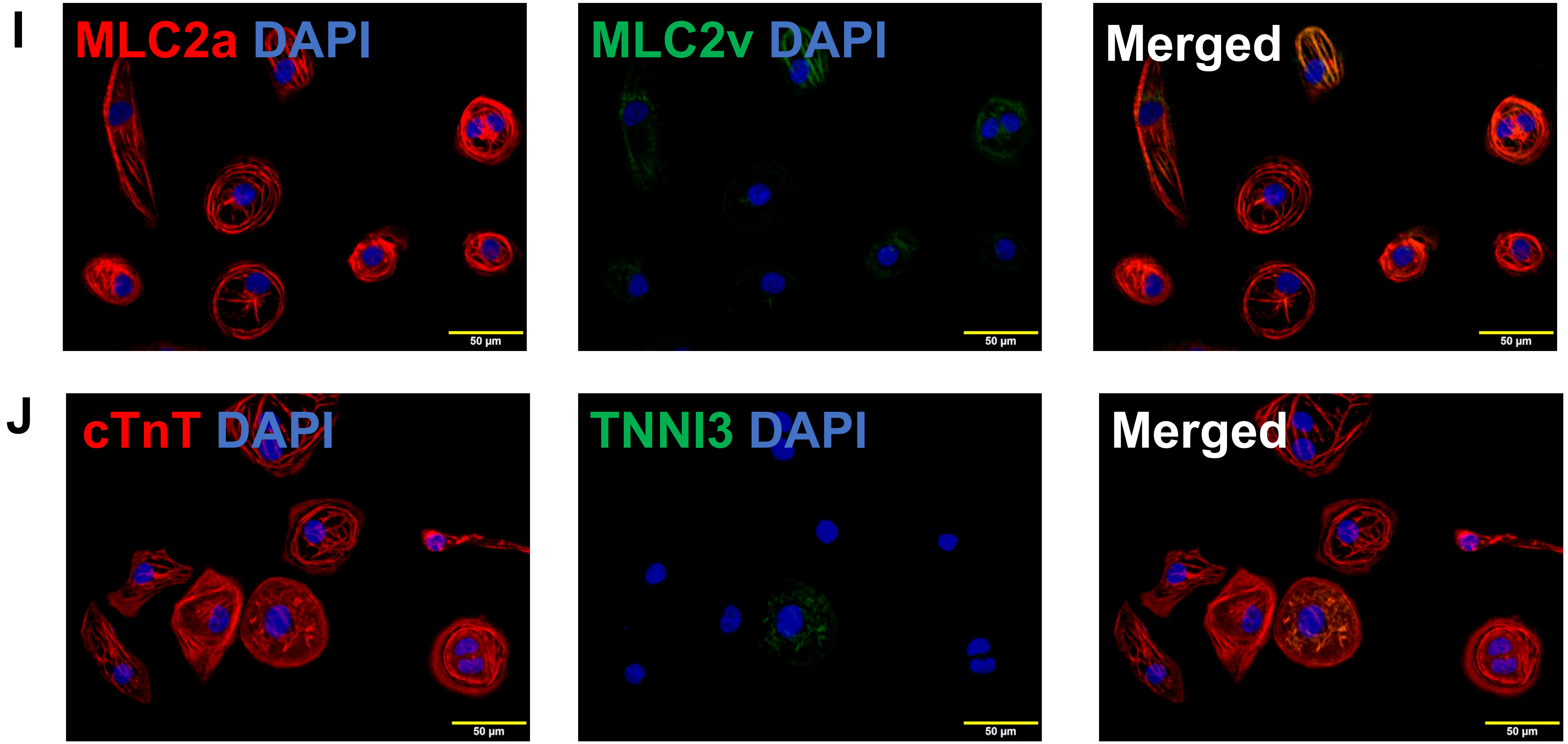

Figure S2. Dual immunostaining of hiPSC-CMs for protein expression of atrial isoform myosin light chain 2 (MLC2a) and ventricular isoform myosin light chain 2 (MLC2v) **(I).** Protein expressions of cardiac troponin T (cTnT) and troponin I isoform 3 (TNNI3) in hiPSC-CMs **(J)**. (Bar = 50 µm).

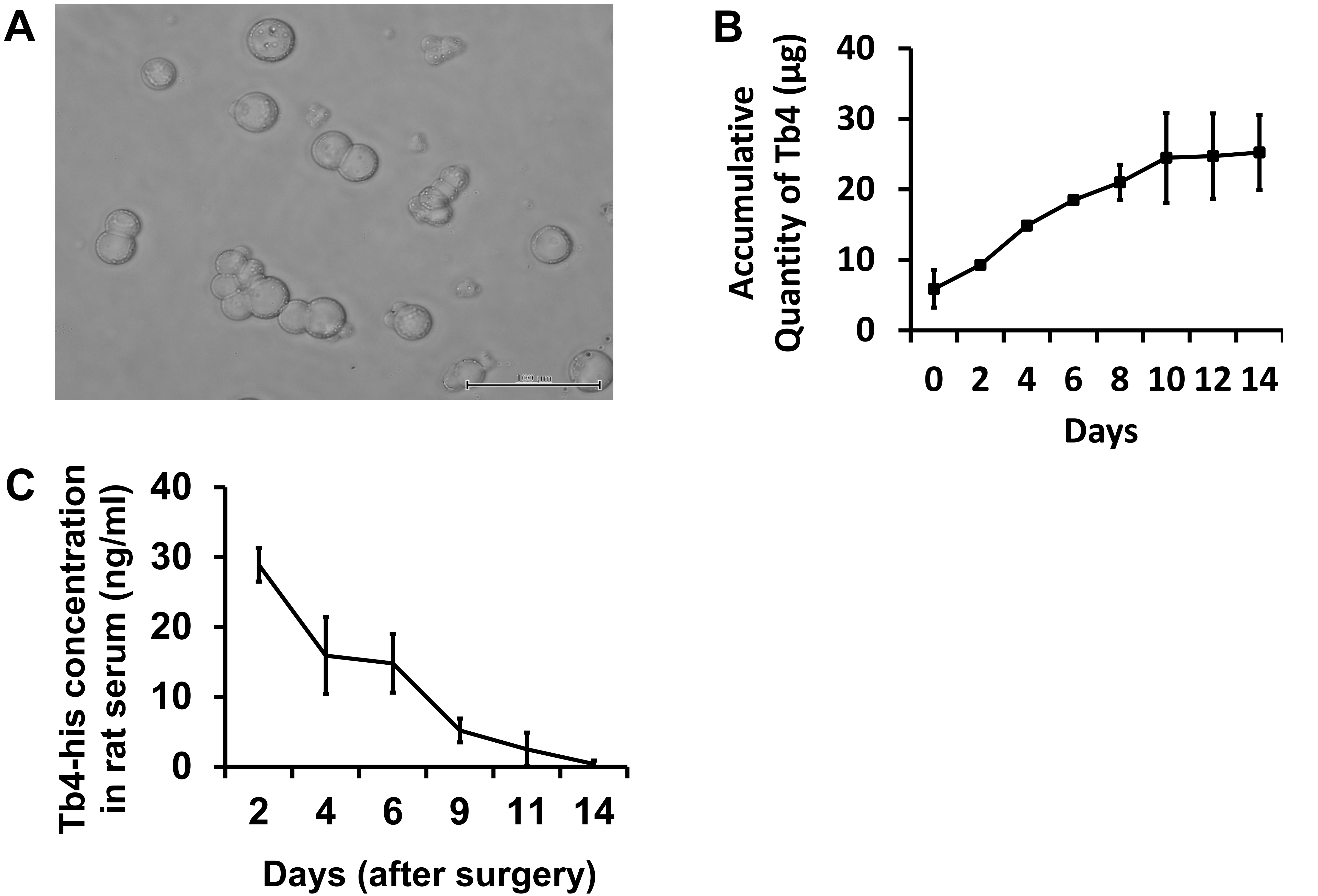

Figure S3. Gelatin microspheres for controlled release of Tb4. **(A)** Typical images of fresh manufactured gelatin microspheres. (Bar = 100 µm). **(B)** Accumulative release profile of Tb4 that was loaded into microspheres up to 14 days *in vitro*. (n=2). **(C)** Tb4-his concentration in rat serum as a function of time *in vivo*. (n=4). Values are presented as the means ± SD.

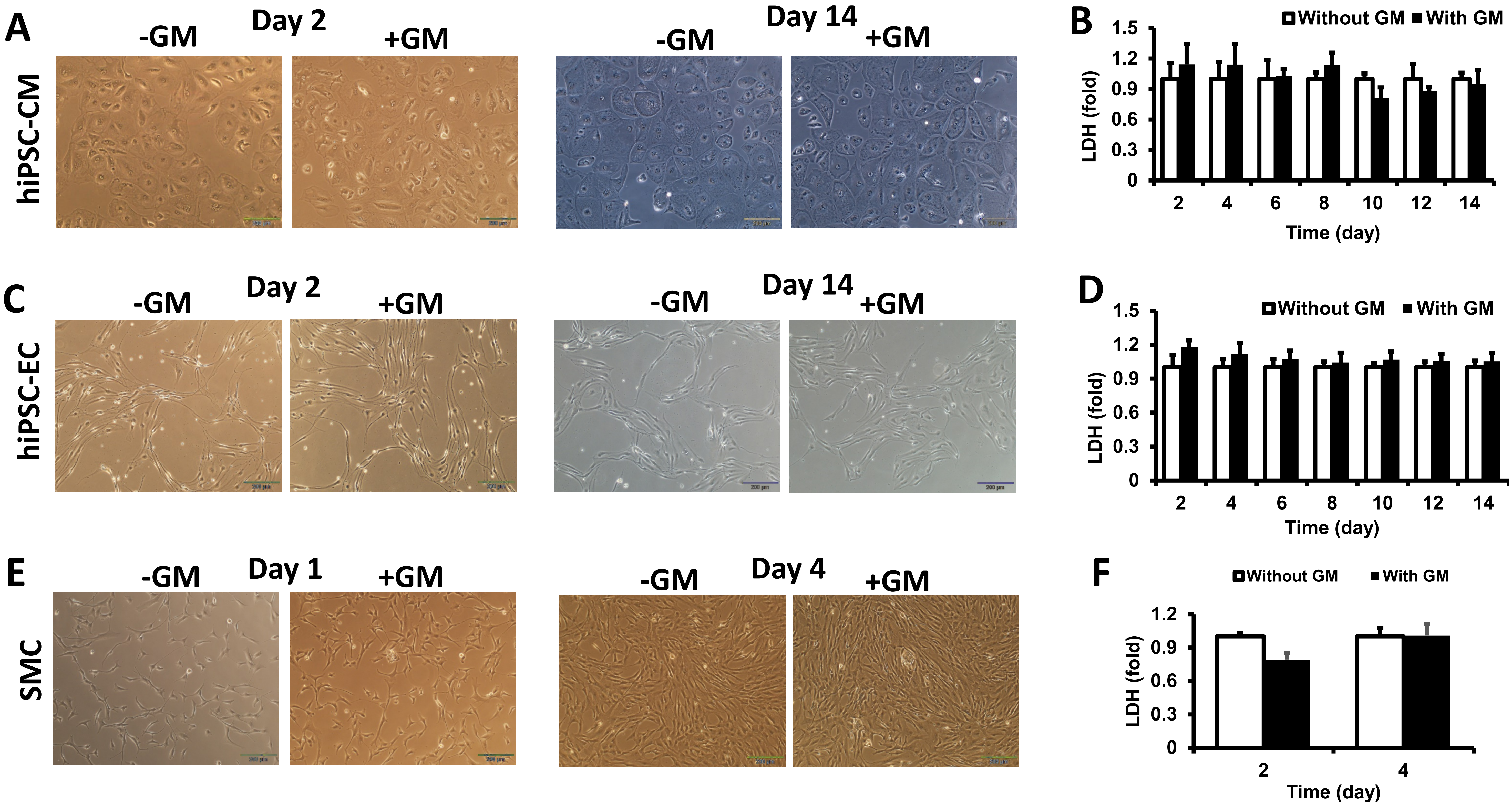

Figure S4. Tests to determine the toxic effect of gelatin microspheres on cardiac cells. (A) Morphological profile of hiPSC-CM treated with or without gelatin microspheres (+GM or –GM). **(B)** Lactate dehydrogenase (LDH) concentration in hiPSC-CM supernatant as a function of time. **(C)** Morphological profile of hiPSC-ECs treated with or without gelatin microspheres. **(D)** LDH concentration in hiPSC-EC supernatant as a function of time. **(E)** Morphological profile of human coronary artery SMCs treated with or without gelatin microspheres. **(F)** LDH concentration in hiPSC-EC supernatant as a function of time. (Bar = 200μm. Values are presented as the means ± SD).

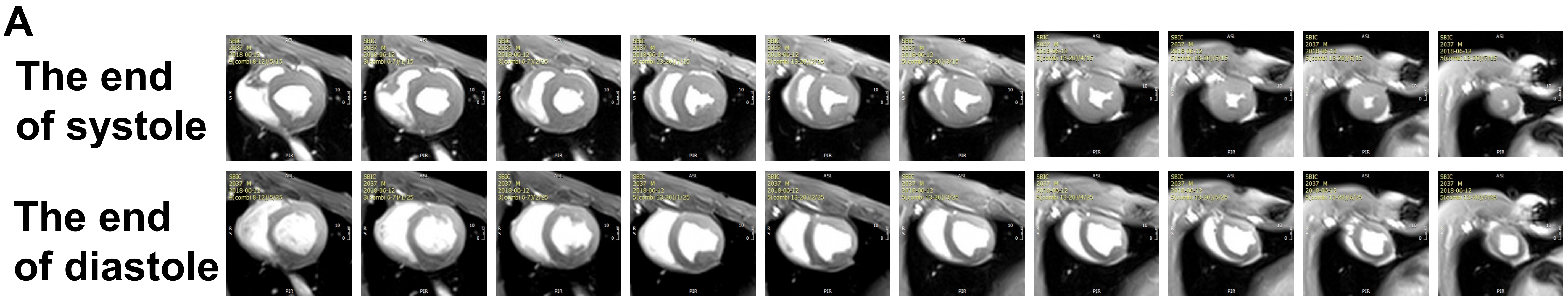

Figure S5. MRI images of pig hearts for measuring left ventricular ejection fraction (LVEF). (A) Representative images of short-axis left ventricular chamber at the end of systole and the end of diastole in a pig heart of the Sham Group.

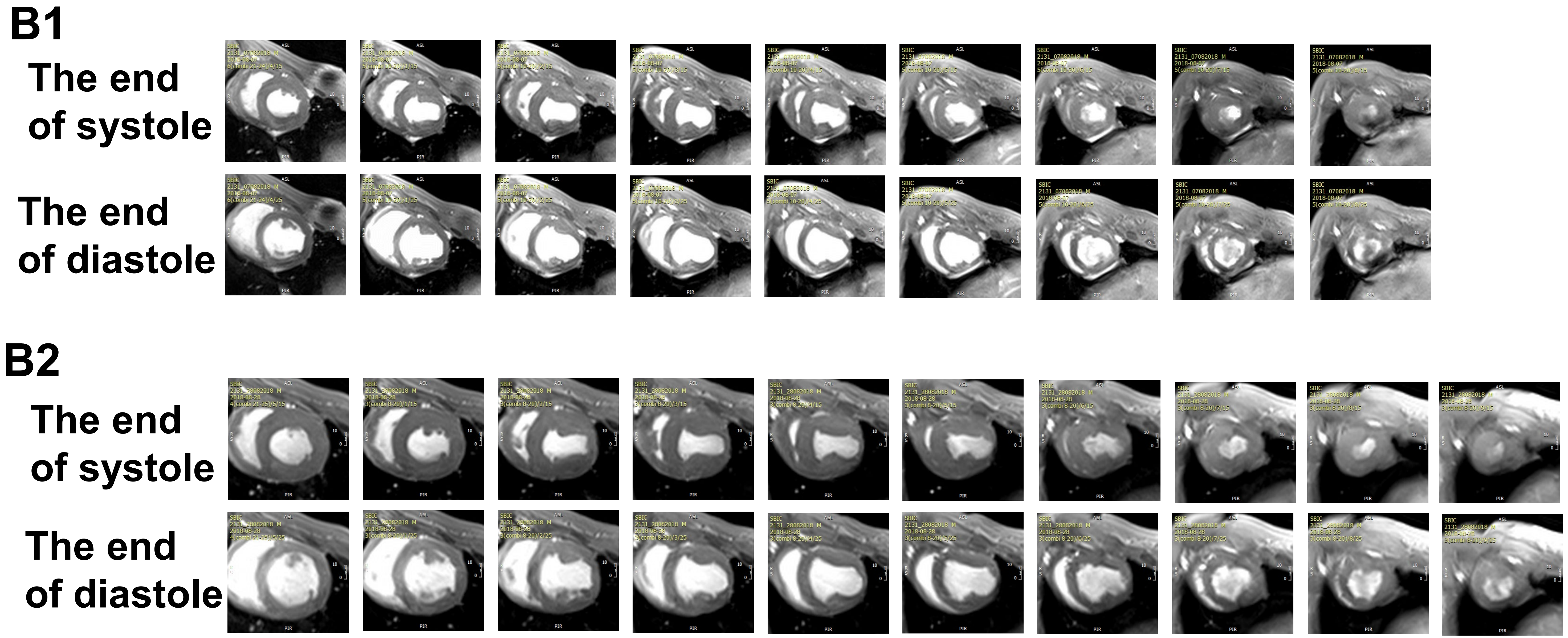

Figures S5. Representative images of short-axis left ventricular chamber at the end of systole and the end of diastole in the pig hearts of the MI Group at weeks-1 **(B1)** and -4 **(B2)** after MI and treatment.

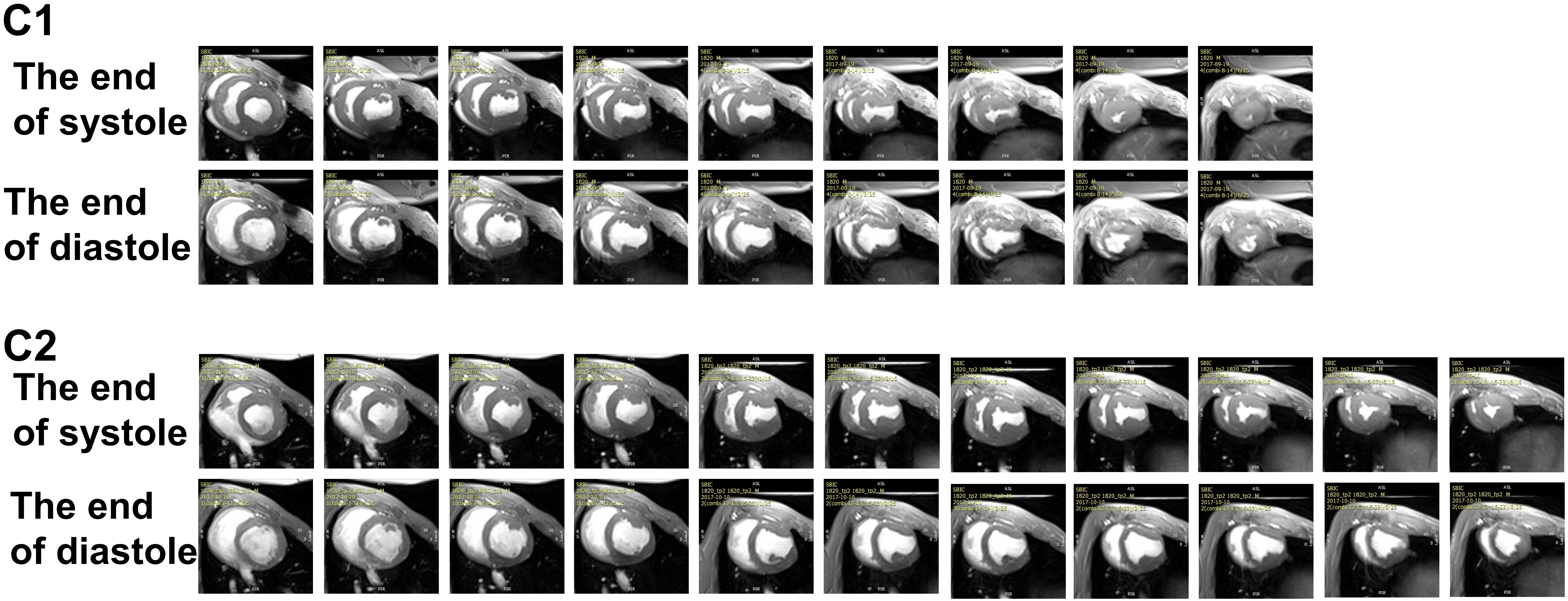

Figures 5C1&C2. Representative images of short-axis left ventricular chamber at the end of systole and the end of diastole in pig hearts of the Tb4 Group at weeks-1 **(C1)** and -4 **(C2)** after MI and treatment.

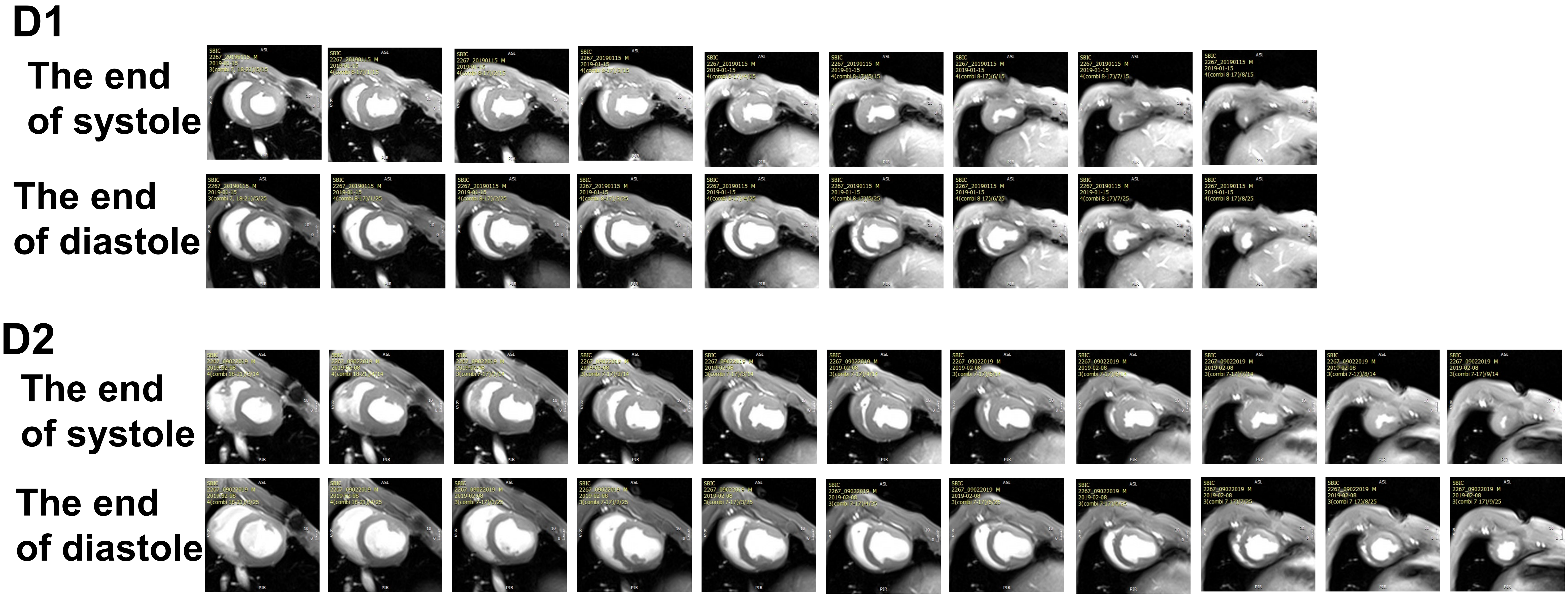

Figures S5. Representative images of short-axis left ventricular chamber at the end of systole and the end of diastole in the pig hearts of the CM Group at weeks-1 (**D1**) and -4 (**D2**) after MI and treatment.

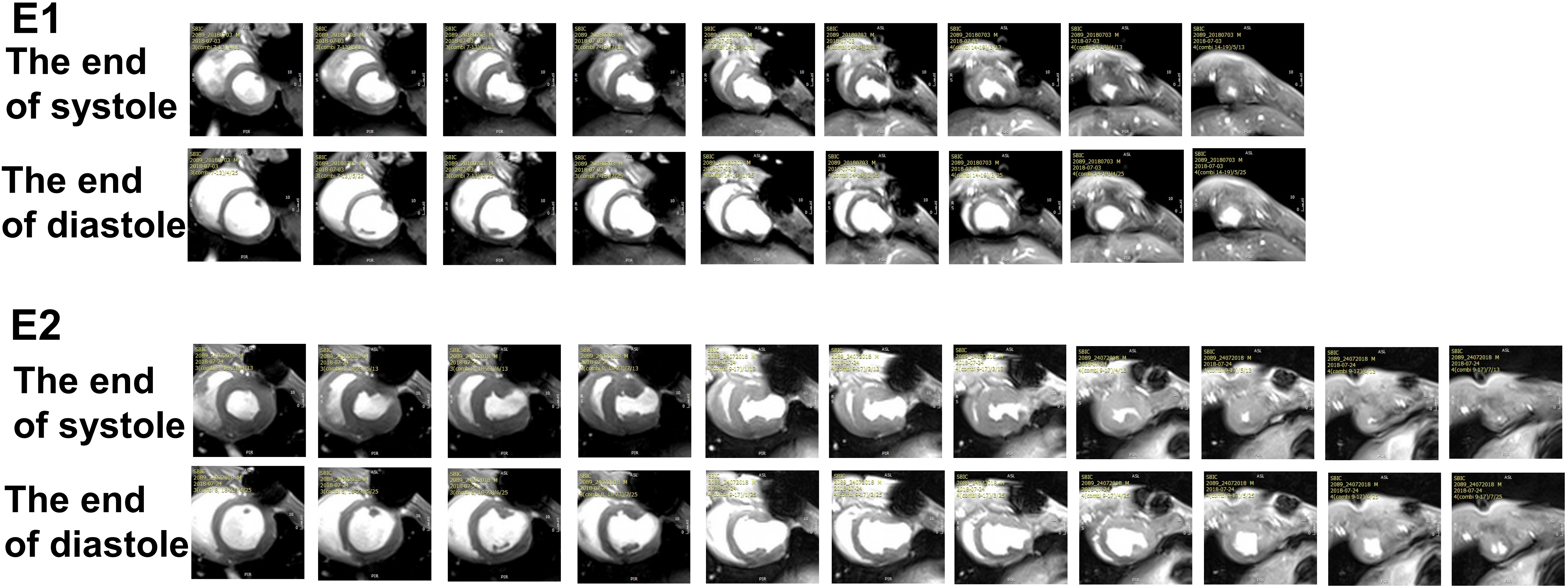

Figures S5. Representative images of short-axis left ventricular chamber at the end of systole and the end of diastole in the pig hearts of the Tb4 + CM Group at weeks-1 (**E1**) and -4 (**E2**) after MI and treatment.

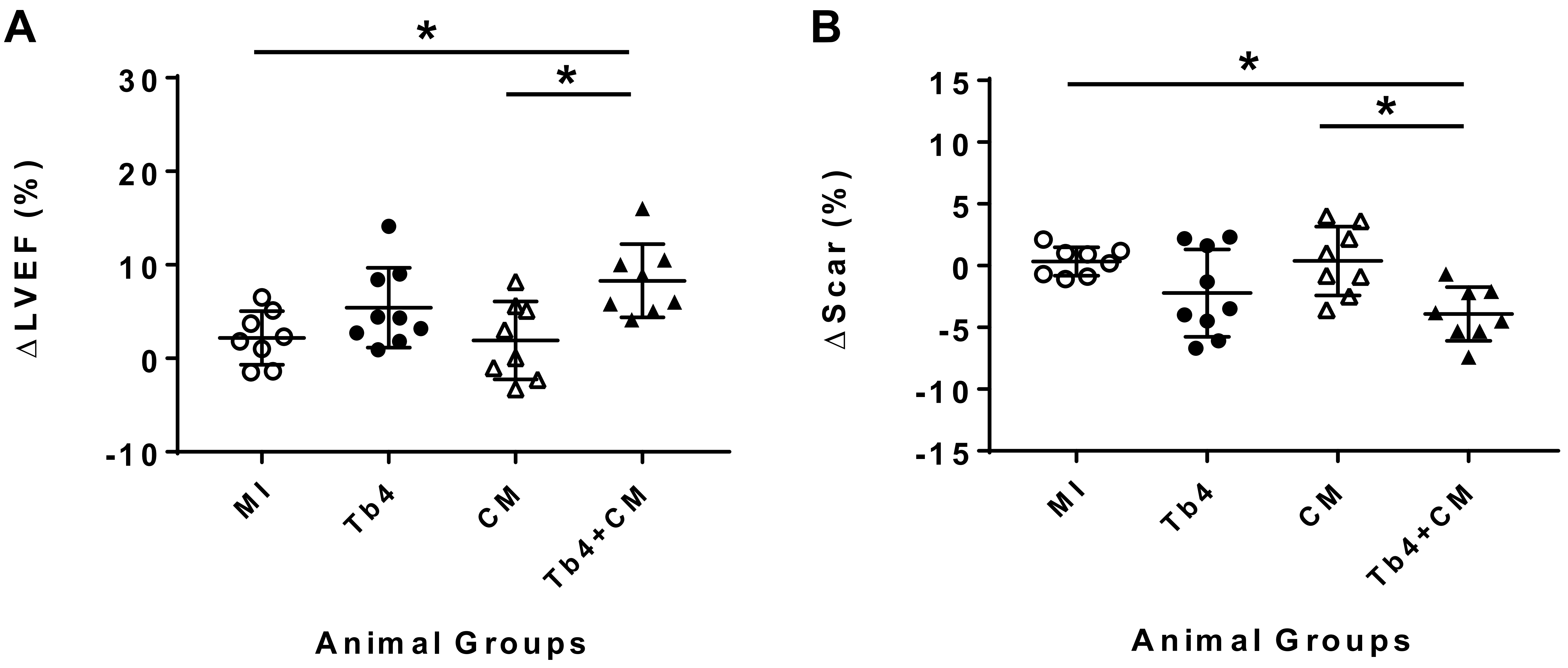

Figure S6. Changes of LVEF (ΔLVEF) and scar area (Δscar) in the pig hearts. (A) ΔLVEF in each group is calculated by LVEF at week-4 minus LVEF at week-1. **(B)** Δscar in each group is calculated by scar area at week-4 minus scar area at week-1. (n = 8 - 9 for each group. One-way ANOVA: *: $P < 0.05$). Values are presented as the means ± SD.

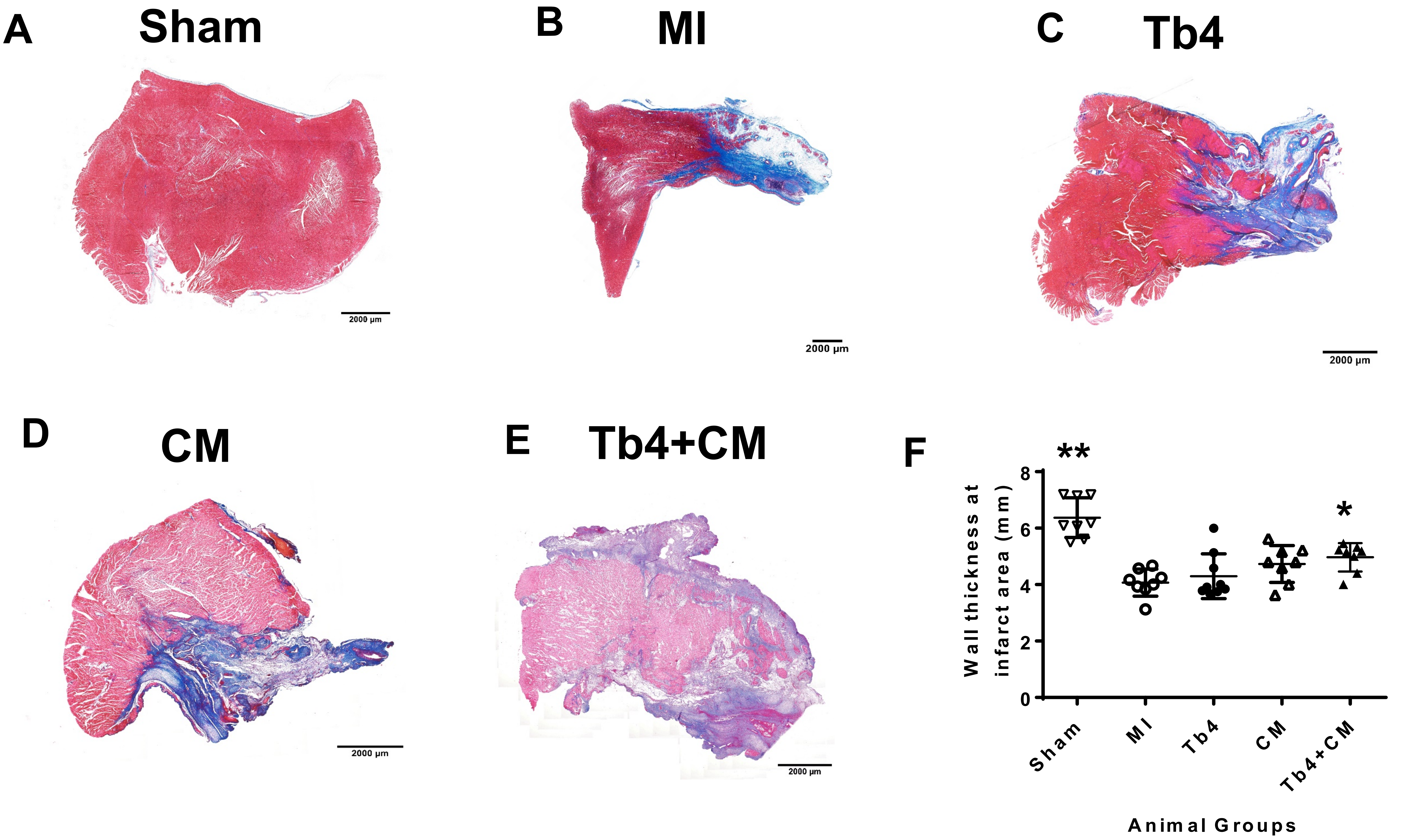

Figure S7. Macro-images of infarct myocardium in the pig hearts using Masson's Trichrome staining. Representative images of Masson's Trichrome stained pig heart tissue to visualize scar in the pig hearts of the Sham **(A)**, MI **(B)**, Tb4 **(C)**, CM **(D)**, and Tb4+CM **(E)** Groups at week-4 after MI and treatment. **(F)** Quantification of wall thickness at infarct area. (n=8 or 9 for each group. One-way ANOVA. **: P < 0.01 vs every other group; *: P < 0.05 vs the MI Group). Values are presented as the means ± SD. Bar = 2000 µm.

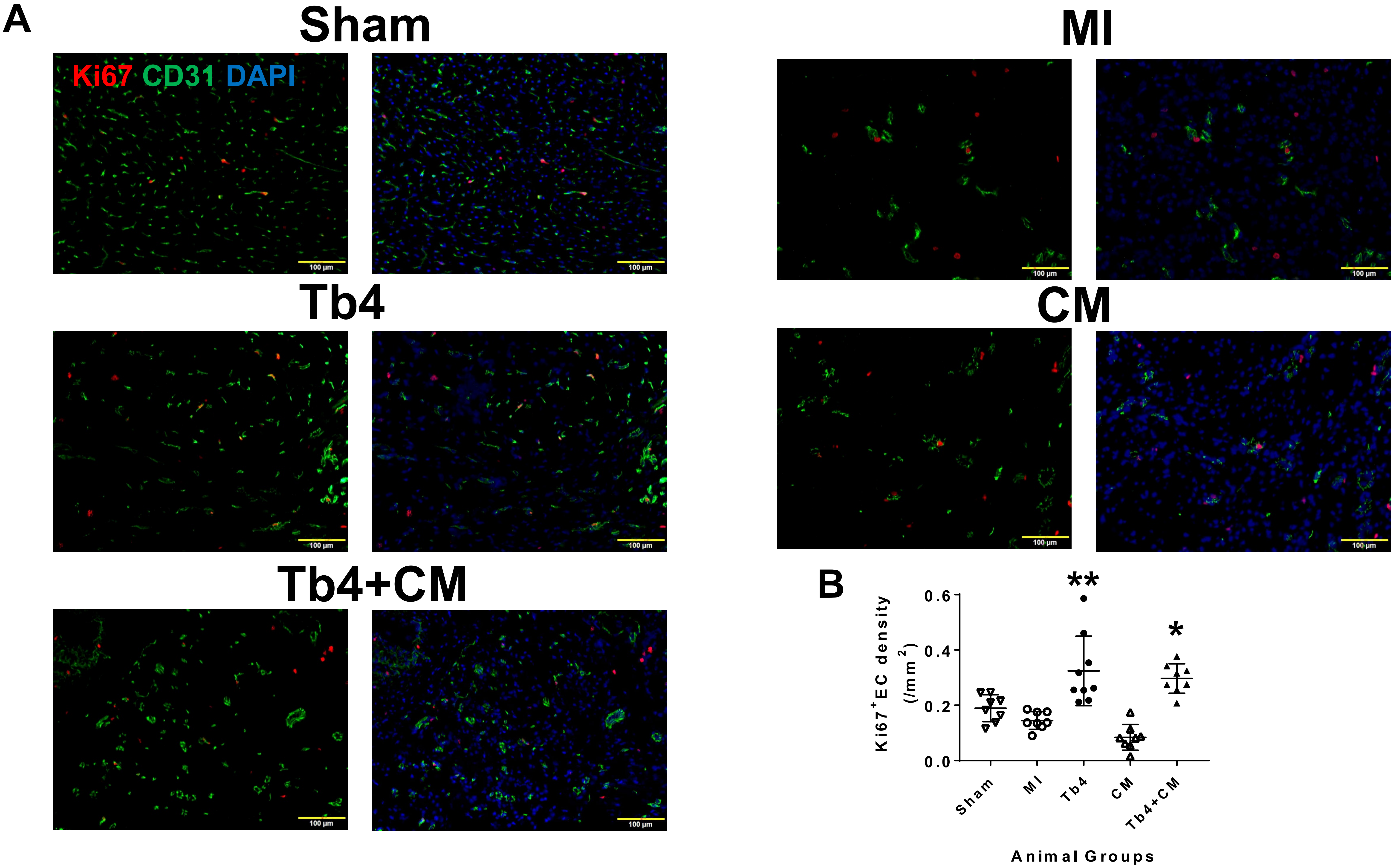

Figures S8. Proliferation of endothelial cells (ECs) and smooth muscle cells (SMCs) in the pig hearts at week-4 after MI and treatment. (A) Representative images of Ki67 expressing ECs in the Sham, MI, Tb4, CM**,** and Tb4 + CM Groups at Week-4 after MI and treatment. **(B)** Quantitative analysis of Ki67^{+}ECs in all animal groups. (n = 8 or 9 for each group. One-way ANOVA. **: $P < 0.01$ vs the Sham, MI, and CM Groups ; *: $P < 0.05$ vs the Sham, MI, and CM Groups). Values are presented as the means ± SD. Bar = 100 µm.

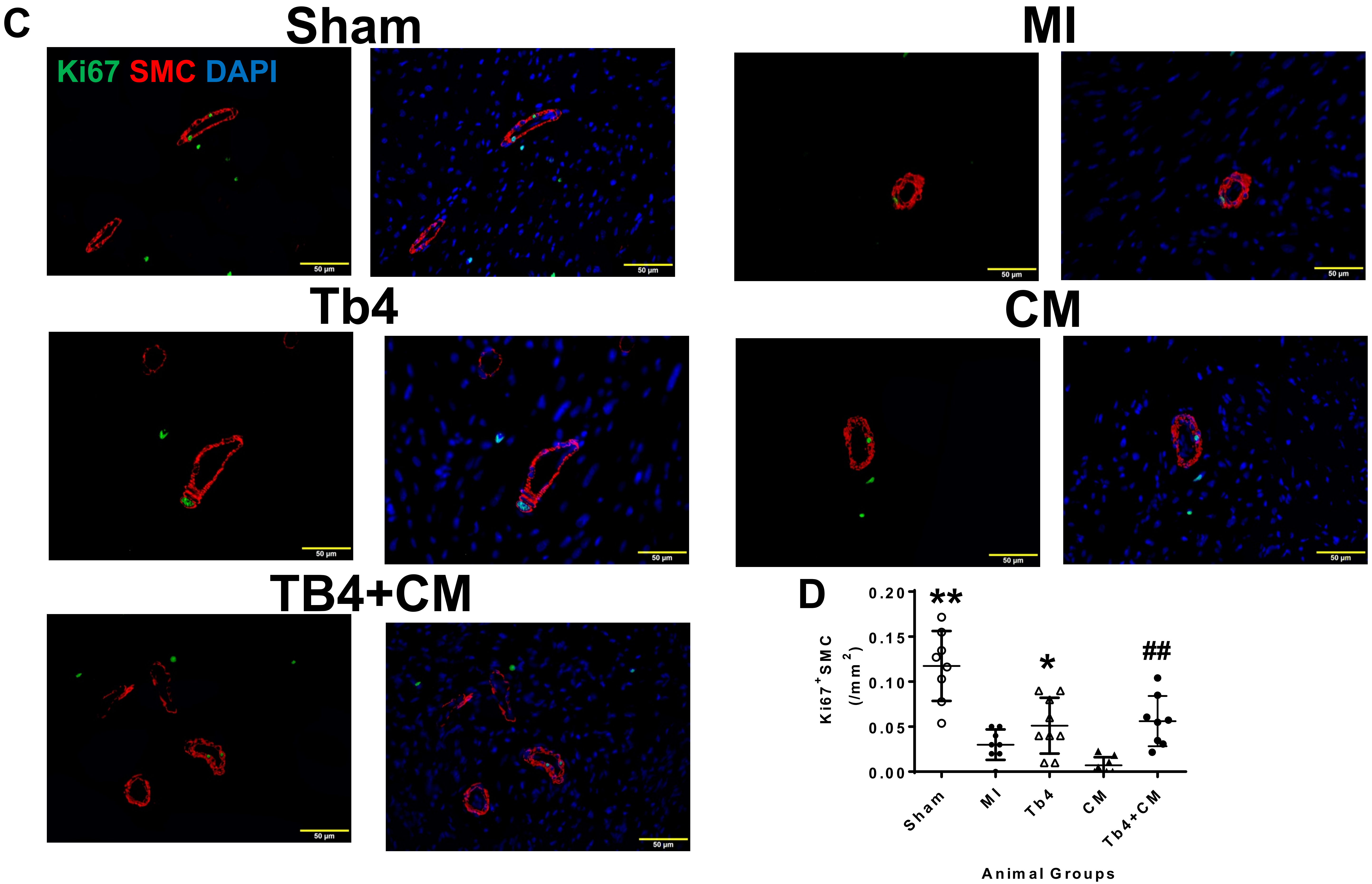

Figures S8. (C) Representative images of Ki67 expressing SMCs in the Sham, MI, Tb4, CM, and Tb4 + CM Groups at Week-4 after MI and treatment. **(D)** Quantitative analysis of Ki67$^+$SMCs in all animal groups. (n = 8 or 9 for each group. One-way ANOVA. **: $P < 0.01$ vs every other group; ##: $P < 0.01$ vs the MI and CM Groups; *: $P < 0.05$ vs the CM Group). Values are presented as the means ± SD. Bar = 50 μm.

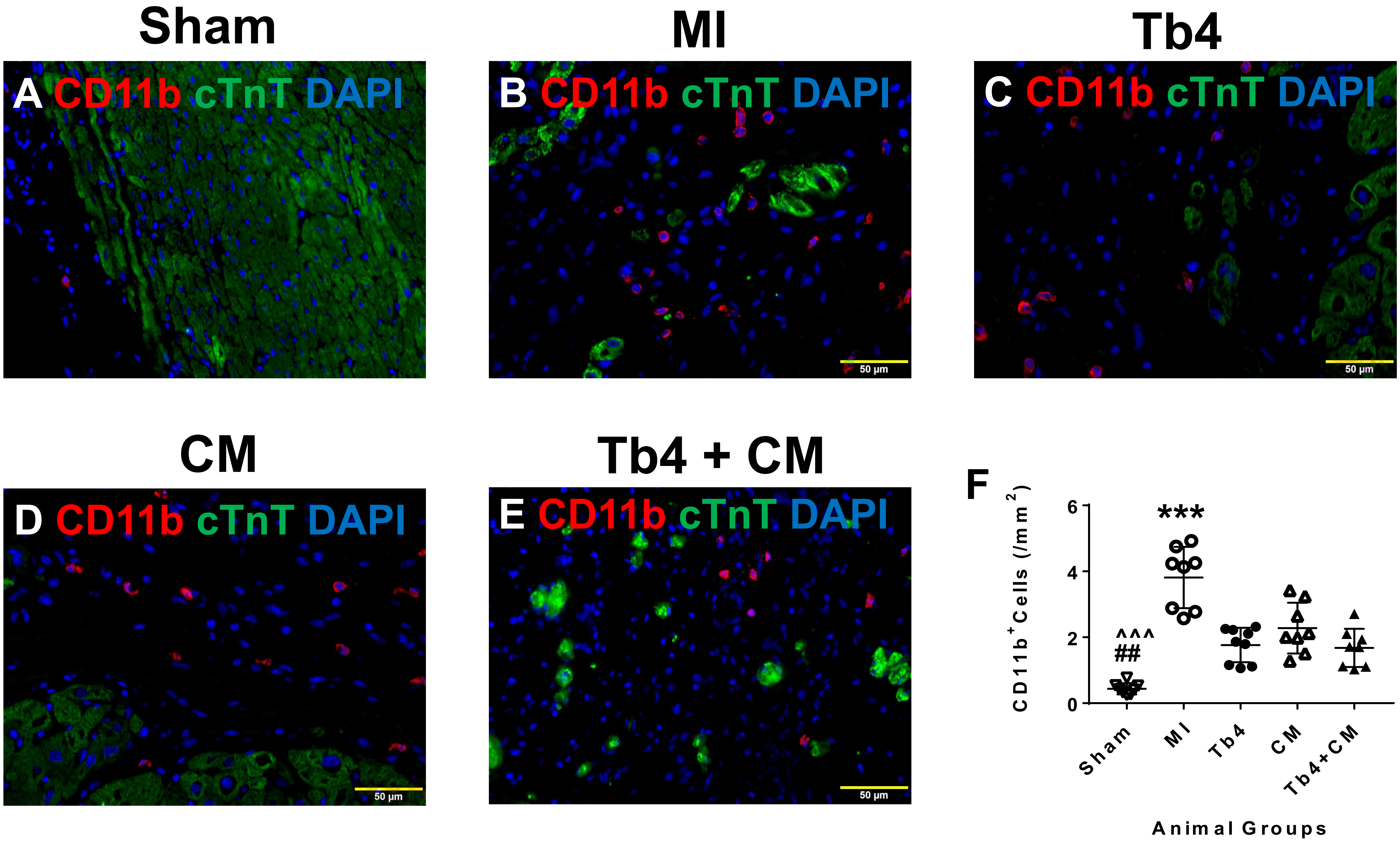

Figure S9. Fluorescence immunostaining for detecting CD11b⁺cells. Representative images of CD11b⁺cells in the Sham **(A)**, MI **(B)**, Tb4 **(C)**, CM **(D)**, and Tb4 + CM **(E)**. **(F)** Quantitative analysis of CD11b⁺cells in all animal groups. (n = 8 or 9 for each group. One-way ANOVA.^^^: $p < 0.001$ vs the MI and CM Groups; ##: $p < 0.01$ vs the Tb4 and Tb4 + CM Groups; ***: $P < 0.001$ vs every other group). Values are presented as the means ± SD. Bar = 50 μm.

Table S1 . Number of animals in each group

	Sham Group	MI Group	Tb4 Group	hiPSC-CM Group	Tb4 + hiPSC-CM Group
**4 weeks**	8	8	9	8	8
**12 weeks**	0	0	0	0	6

Table S2. Human Angiogenesis Array Q2

	hiPSC-CM	hiPSC-CM + Tb4
**Activin A**	30.16	21.90
**AgRP**	2.88	3.31
**Angiogenin**	1.34	1.30
**ANG-2**	1.00	10.17
**ANGPTL4**	0	0
**bFGF**	0	22.48
**ENA-78**	17.51	2.00
**GRO**	0	3.55
**HB-EGF**	0	0.35
**HGF**	0	0
**IFNg**	0.25	0
**IGF-I**	51.46	0
**IL-1a**	0	0
**IL-2**	3.04	1.26
**IL-6**	0.86	0
**IL-8**	0	0
**IL-17**	0	0
**IP-10**	0.29	0
**Leptin**	18.66	18.45
**LIF**	9.21	12.81
**MCP-1**	54.95	58.22
**PDGF-BB**	1.62	1.28
**PIGF**	0.73	2.48
**RANTES**	0	0.15
**TGFb1**	0	0
**TIMP-1**	7497.31	6579.71
**TIMP-2**	3233.89	2480.30
**TNFa**	0	0
**TNFb**	0	0
**TPO**	104.52	75.75

Table S3. Human Angiogenesis Array Q3

	hiPSC-CM	hiPSC-CM + Tb4
**ANG-1**	0	0
**Angiostatin**	4554.59	4568.78
**CXCL16**	1.37	0
**EGF**	0.12	0.05
**FGF-4**	25.98	151.24
**Follistatin**	7217.05	8028.29
**G-CSF**	0	0.44
**GM-CSF**	0.37	2.07
**I-309**	0	0
**IL-1b**	0	2.90
**IL-4**	0.28	1.16
**IL-10**	0	0
**IL-12p40**	9.56	10.38
**IL-12p70**	0.20	0.47
**I-TAC**	2.23	9.41
**MCP-2**	1.41	0.33
**MCP-3**	1.39	1.46
**MCP-4**	2.00	22.86
**MMP-1**	218.17	94.03
**MMP-9**	4.61	0
**PECAM-1**	25.08	2.27
**TGFa**	11.69	8.21
**TGFb3**	0.20	0.45
**Tie-1**	106.91	0
**Tie-2**	0	5.25
**uPAR**	309.60	197.61
**VEGF**	87.75	123.16
**VEGF R2**	7.74	2.44
**VEGF R3**	9.35	8.19
**VEGF-D**	9.51	0

Video-1: shows the upper abdomen to the pelvis, including part of the liver, small and large intestines, kidneys and urinary bladder at 1 week post cell implantation.

Video-2: shows the neck (larynx) to the upper abdomen, including the heart, lung, liver, gallbladder, stomach and part of the large bowel at 1 week post cell implantation.

Video3: shows the head (brain) to the upper chest at 1 week post cell implantation.

Video-4: shows Sagittal view of the thorax centered at the level of the heart at 1 week post cell implantation.

Video-5: shows Head and upper neck including the brain, larynx at 3 months post-cell implantation.

Video-6: shows upper neck (larynx) to the upper chest (mediastinum)_ larynx, lungs, heart at 3 months post-cell implantation.

Video-7: shows the upper chest (mediastinum) to the upper abdomen, including the heart, lungs, part of the liver and gallbladder at 3 months post-cell implantation.

Video-8: shows the upper abdomen to the abdomen, liver, gallbladder, bowels, kidneys and urinary bladder at 3 months post-cell implantation.

Video-9: shows the abdomen to the pelvis - bowels, kidneys and urinary bladder at 3 months post-cell implantation.

Video-10: shows the lower pelvis, perineum area with partly visualised urinary bladder, rectum and part of the vagina at 3 months post-cell implantation.
